# IL-17 induces NSCLC cell migration and invasion by elevating MMP19 gene transcription and expression through the interaction of p300-dependent STAT3-K631 acetylation and its Y705-phosphorylation

**DOI:** 10.32604/or.2023.031053

**Published:** 2024-03-20

**Authors:** WEN GE, YA LI, YUTING RUAN, NINGXIA WU, PEI MA, TONGPENG XU, YONGQIAN SHU, YINGWEI WANG, WEN QIU, CHENHUI ZHAO

**Affiliations:** 1 Department of Immunology, Nanjing Medical University, Nanjing, 210000, China; 2 Key Laboratory of Immunological Environment and Disease, Nanjing Medical University, Nanjing, 210000, China; 3 Department of Oncology, The First Affiliated Hospital of Nanjing Medical University, Nanjing, 210000, China; 4 Jiangsu Key Laboratory of Cancer Biomarkers, Prevention and Treatment, Collaborative Innovation Center for Cancer Personalized Medicine, Nanjing Medical University, Nanjing, 210000, China

**Keywords:** NSCLC cell migration and invasion, IL-17, p300, STAT3, MMP19, Acetylation and phosphorylation

## Abstract

The cancer cell metastasis is a major death reason for patients with non-small cell lung cancer (NSCLC). Although researchers have disclosed that interleukin 17 (IL-17) can increase matrix metalloproteinases (MMPs) induction causing NSCLC cell metastasis, the underlying mechanism remains unclear. In the study, we found that IL-17 receptor A (IL-17RA), p300, p-STAT3, Ack-STAT3, and MMP19 were up-regulated both in NSCLC tissues and NSCLC cells stimulated with IL-17. p300, STAT3 and MMP19 overexpression or knockdown could raise or reduce IL-17-induced p-STAT3, Ack-STAT3 and MMP19 level as well as the cell migration and invasion. Mechanism investigation revealed that STAT3 and p300 bound to the same region (−544 to −389 nt) of MMP19 promoter, and p300 could acetylate STAT3-K631 elevating STAT3 transcriptional activity, p-STAT3 or MMP19 expression and the cell mobility exposed to IL-17. Meanwhile, p300-mediated STAT3-K631 acetylation and its Y705-phosphorylation could interact, synergistically facilitating MMP19 gene transcription and enhancing cell migration and invasion. Besides, the animal experiments exhibited that the nude mice inoculated with NSCLC cells by silencing p300, STAT3 or MMP19 gene plus IL-17 treatment, the nodule number, and MMP19, Ack-STAT3, or p-STAT3 production in the lung metastatic nodules were all alleviated. Collectively, these outcomes uncover that IL-17-triggered NSCLC metastasis involves up-regulating MMP19 expression via the interaction of STAT3-K631 acetylation by p300 and its Y705-phosphorylation, which provides a new mechanistic insight and potential strategy for NSCLC metastasis and therapy.

## Introduction

Non-small cell lung cancer (NSCLC) is common type of malignancy, and the leading death cause of NSCLC patients is associated with the cancer metastasis [1–3]. Recently, accumulating evidence has demonstrated that NSCLC is an inflammation-related cancer [4–6], and the overexpression of inflammatory cytokines or mediators in NSCLC microenvironment can induce the cancer cell proliferation, migration and invasion [7–10], but the precise mechanism, e.g., IL-17-mediated NSCLC metastasis has not been characterized.

IL-17A, also known as IL-17, is a pro-inflammatory cytokine [10]. Many scholars have reported that the expression increase of IL-17 in the cancer tissues can improve cell proliferation resulting in cancer growth [11,12] or elevating MMP2 and MMP9 production leading to NSCLC metastasis [13]. It is recognized that cancer cell metastasis is a complex process that involves the extracellular matrix (ECM) degradation and basement membrane penetration [14]. Several studies have demonstrated that MMP1, MMP2, MMP9, MMP13 and MMP19 in some cancer tissues such as NSCLC or gastric cancer are all raised, and these MMP members can degrade basement membrane and open the channel for cancer’s invasion and metastasis [13,15–17]. In the early stage of our experiments, we found that MMP19 expression was greatly higher than other MMP members both in the detected NSCLC tissues and in the NSCLC cells stimulated with IL-17. However, little is known about how IL-17 can induce MMP19 expression causing NSCLC metastasis.

Signal transducer and activator of transcription 3 (STAT3) is a member of STAT family, and can activate its target gene transcription in many cancer cells upon various stimulus inducing the change of cell behaviors [18–20]. For example, the STAT3 activated by IL-17 or IL-6 can facilitate cancer cell angiogenesis, migration, invasion and metastasis via increasing the expression of vascular endothelial growth factor (VEGF), O-GlcNacylation, MMP or the switch of epithelial-mesenchymal transition (EMT) to mesenchymal-epithelial transition (MET) [19–23]. Our previous test of this work has found that the NSCLC cells under IL-17 stimulation can markedly activate STAT3 and up-regulate MMP19 expression, but their relationship and effects of STAT3 activation on MMP19 induction in the NSCLC cells exposed to IL-17 are not clear.

Current documents have reported that the protein post-translational modification (PTM), such as phosphorylation or acetylation is essential for regulating protein function and cell behaviors [23–26]. Previous researches have revealed STAT3 can subject to phosphorylation (e.g., p-STAT3 at Ser727 or Tyr705) and acetylation, and its phosphorylation or acetylation is necessary for STAT3 activation promoting cancer tumorigenesis or metastasis and STAT3-driven gene transcription in the microglia and other cancers [23,27,28], but the role of STAT3 acetylation and the interaction of STAT3 acetylation and phosphorylation in MMP19 expression as well as NSCLC cell metastasis in response to IL-17 remain undefined.

p300 is a transcriptional co-activator with histone acetyltransferase (HAT) activity [29], and p300 can acetylate the lysine of histones or transcription factors resulting in cell behavior alteration [29–32]. Cai et al. [31] have confirmed that p300 can epigenetically regulate hepatocellular carcinoma (HCC) progression through acetylating metabolic enzymes, and Hou et al. [32] have pointed out that p300 can enhance NSCLC cell proliferation, migration and invasion via inducing EMT. Given that CREPT/RPRD1B promotes tumorigenesis by STAT3-driven gene transcription in p300-dependent manner [28], p300 augments the cell metastasis of HCC and NSCLC [31,32], and p300 overexpression is also proved in our detected NSCLC samples, whether p300 can acetylate STAT3 and affect its-Y705 phosphorylation, and the effect of the interaction between these two posttranslational modifications on MMP19 expression, cell migration and invasion in IL-17-stimulated NSCLC cells require to be ascertained.

In the present study, we first examined the expression of IL-17 receptor A (IL-17RA, a major receptor), p300, Y705-phosphorylated STAT3 (i.e., p-STAT3), acetylated STAT3 (Ack-STAT3) and MMP19 both in NSCLC tissues and in the NSCLC cells stimulated by IL-17. Then, we explored *in vitro* that the role and mechanism of p300, STAT3, MMP19 expression as well as p300-mediated STAT3 acetylation or its phosphorylation and their interaction in regulating MMP19 gene transcription and expression, cell migration and invasion in the NSCLC upon IL-17. Moreover, we utilized nude mouse metastasis model to evaluate that the influence of silencing p300, STAT3 or MMP19 gene plus IL-17 treatment on NSCLC metastasis and related protein expression.

## Materials and Methods

### Reagents, antibodies and plasmids

Recombinant human IL-17A was provided by R&D systems (Tustin, CA, USA). TRIzol and lipofectamine 2000 were from Invitrogen (Carlsbad, CA, USA). The reverse transcription reagent 2×Hifair II SuperMix plus was from Yeasen Biotechnology (Shanghai, China). PCR reagent 2×Taq Master Mix (Dye Plus), reverse transcription reagent kit, SYBR-Green master mix and DL2000 DNA marker were from Vazyme Biotech (Nanjing, China). Dual-luciferase reporter assay system kit was obtained from Promega (Madison, WI, USA). ChIP kit was purchased from Millipore (Bedford, MA, USA). Cytoplasmic and nuclear protein extraction kit (P0028) was supplied by Beyotime (Nanjing, China). Tip60 inhibitor TH1834 and p-STAT3-Y705 inhibitor Stattic were from MedChemExpress (Monmouth Junction, NJ, USA). p300 or GCN5 inhibitor (C646 or MB-3) was provided by CSN pharma (Shanghai, China) or Santa Gruz Biotechnology (Dallas, TX, USA). MycoAlert™ mycoplasma detection kit was purchased from Lonza Biologics (Portsmouth, NH, USA). Anti-IL-17RA antibody (Ab) was from Thermo Fisher Scientific (Waltham, MA, USA). Anti-p300, STAT1-6, p-STAT1-6 and acetylated-lysine (Ack) Abs were from Cell Signaling Technology (Danvers, MA, USA). Anti-MMP19 Ab was provided by ABclonal (Wuhan, China). Anti-HA (561) Ab was from MBL (Nagoya, Japan). Anti-β-actin (AF0003) and Lamin B1 (AF1408) Abs were from Beyotime (Shanghai, China). HRP-anti-rabbit IgG (BS13278) Ab was supplied by Bioworld Technology (Nanjing, China). pcDNA3.1/p300 and pCMV/STAT3 plasmids were obtained from Hewu Biotechnology Co., Ltd. (Shanghai, China) or Public Protein/Plasmid Library (Nanjing, China). SiIL-17RA, shp300, shSTAT3 and shMMP19 plasmids were from GenePharma (Shanghai, China). pIRES2/MMP19 overexpression or STAT3(K631R), STAT3(Y705F) and STAT3(Y705D) mutation plasmids were constructed by General Biosystems (Chuzhou, China).

### Human specimens and cell lines

The cancer tissues and adjacent normal tissues of 10 NSCLC patients were from Jiangsu Cancer Hospital. The patients suffering from other diseases, e.g., infection, autoimmune, inflammatory diseases and other cancers were excluded. This study was approved by the Ethics Committee of the First Affiliated Hospital of Nanjing Medical University (2019-SRFA-052) and performed in compliance with the Declaration of Helsinki. Informed consent for this procedure was obtained from each participant. The NSCLC tissue arrays (*n* = 52, paired) were provided by Outdo Biotech Co., Ltd. (Shanghai, China). The human NSCLC cell lines such as NCI-H1299 (RRID: CVCL_0060), NCI-H1975 (RRID: CVCL_1511) or PC-9 (RRID: CVCL_B260), and normal bronchial epithelium cell line BEAS-2B (RRID: CVCL_0168) were purchased from American Type Culture Collection (ATCC) or European Collection of Authenticated Cell Cultures (ECACC). The above-mentioned cell lines were authenticated via short-tandem repeat (STR) analysis by General Biosystems. Besides, all cell lines used in the experiments were routinely tested for mycoplasma by using MycoAlert™ mycoplasma detection kit and showed mycoplasma-free.

### Immunohistochemical (IHC) staining

The tissue microarrays and paired adjacent tissues from 52 NSCLC patients were incubated with anti-IL-17RA, anti-p300, anti-p-STAT3 and anti-MMP19 Abs, respectively, and then incubated with HRP-anti-rabbit IgG for 1 h followed by DAB reaction. The IHC staining score was calculated by the staining intensity (negative, 0; weak, 1; moderate, 2; strong, 3) and staining range (i.e., percentage of positive cells, 0%, 0; 1%–25%, 1; 26%–50%, 2; 51%–75%, 3; and 76%–100%, 4). The final score was calculated by the intensity and positive rate scores, namely, 0–1, negative expression; 2–4, weak positive expression; 6–12, strong positive expression.

### Cell culture and IL-17 stimulation

Cells were maintained with 10% fetal bovine serum (FBS) added Dulbecco’s Modified Eagle’s Medium (DMEM). For IL-17 stimulation, the NSCLC cell lines were suffered from 12 h serum starvation, and then were given 0, 25, 50 and 100 ng/ml IL-17 for different time.

### RT-PCR and real-time PCR

Total RNA from NSCLC tissues or cells was isolated using TRIzol. The cDNA was produced and RT-PCR assay was carried out. Besides, real-time PCR was done and the results were normalized to β-actin and analyzed using the 2^−ΔΔCt^ method. RT-PCR and real-time PCR primers are shown in Suppl. Tables S1 and S2.

### Co-immunoprecipitation (Co-IP)

NSCLC tissues or cells extracts were incubated with protein G-Sepharose beads and pre-immune IgG. After centrifugation, the supernatant was incubated with 2 μg of relevant Ab overnight. Thereafter, the protein G-Sepharose was added and incubated for 2 h. The precipitates were washed and eluted with 2 × SDS-PAGE sample buffer, and then analyzed by immunoblotting (IB).

### IB experiment

Total protein from the tissues and cells was extracted by RIPA cell lysis buffer, and the proteins (30 μg/lane) were separated by SDS-PAGE and transferred onto PVDF membrane. Afterwards, the membrane was incubated with primary Ab overnight followed by HRP-conjugated corresponding Ab for 1 h. The chemiluminescent substrate was applied to the blots, and the signals were captured by ChemiDoc Imaging System (Bio-Rad, Hercules, CA, USA). Autoradiograms were quantified by densitometry with the Quantity One software (Bio-Rad, Hercules, CA, USA).

### Cytoplasm and nucleus extraction

The cytoplasmic and nuclear protein in 8 × 10^5^ H1299 cells was extracted using the corresponding extraction kit according to the manufacturer’s instruction. The extracted proteins were examined by IB test.

### Wound healing assay

NSCLC cells (1 × 10^5^/well) were incubated for 48 h, and a 100 μL pipette tip was used to scratch the monolayer and create a constant gap. The detached or dead cells were washed away with PBS, and then the medium with 50 ng/ml IL-17 was added. The movement of cells filling the gap was recorded by photographing at 0 and 24 h. The images were analyzed by ImageJ software.

### Cell migration or invasion assay

For migration, cells (5 × 10^4^/well) were seeded in top chambers of the Transwell plates in the FBS-free media with membrane inserts without Matrigel coated, and the complete media were added to the wells (lower compartment), followed by incubation for 24 h. The cells that migrated to the lower membrane were fixed and stained with 0.1% crystal violet for 30 min and counted. For invasion, the upper compartment of the insert was coated with Matrigel and the cells on the lower side of the insert membrane were fixed and stained.

### Mass spectrometry (MS) detection

H1299 cells (8 × 10^5^) were transfected with HA-STAT3 plasmids for 48 h plus IL-17 (50 ng/mL) for 3 h. The anti-HA immunoprecipitants were subjected to SDS-PAGE and silver staining. The protein bands were collected. MS detection was performed at Center of Hygienic Analysis and Detection of Nanjing Medical University, Nanjing, China.

### Plasmid construction and cell transfection

The plasmids of pGL3-MMP19 full length (FL, −1163 to +313 nt) promoter, and the four truncated promoters (−662 to +313 nt, −312 to +313 nt, −104 to +313 nt, +62 to +313 nt) were constructed by inserting these fragments of MMP19 promoter into pGL3-basic vector respectively. The primer sequences of pGL3-MMP19-FL and the truncated promoters were listed in Table S3. For plasmid transfection, the cells were incubated with the mixture of 2 µg plasmids and 4 µl lipofectamine 2000 for 48 h, and then the mixture was washed away.

### LV-shRNA preparation and infection

The most effective shp300, shSTAT3 and shMMP19 plasmids were packaged with lentiviruses (LV) to prepare LV-shp300, LV-shSTAT3 and LV-shMMP19. Next, H1299 cells (2 × 10^5^) were infected with LV-shRNA at the titer of 1 × 10^7^ TU/ml for 48 h, and the cells stably infected by the LV-shRNA were selected with 2 μg/mL puromycin.

### Luciferase reporter assay

pGL3-MMP19-FL and four truncated MMP19 promoter plasmids were separately transfected into H1299 cells with pRL-SV40 for 48 h. Then, the cells were treated with or without 50 ng/mL IL-17 for 3 h, and MMP19 promoter activity was determined by dual-luciferase reporter kit.

### ChIP, re-ChIP and chromatin immunodepletion (ChID) assay

ChIP assay was carried out by using anti-preimmune IgG, STAT3 or p300 according to the protocol from the manufacturer. For re-ChIP, the primary ChIP production was immunoprecipitated again with another ChIP-grade Abs, and the immunoprecipitated DNA was amplified by RT-PCR and real-time PCR. For ChID, the second-round immunoprecipitation was done with anti-Ack Ab, and MMP19 promoter fragment immunodepleted with anti-Ack in the supernatant was measured by real-time PCR. The ChIP-PCR primers for MMP19 promoter fragment were exhibited in Suppl. Table S2.

### Metastatic model experiment

Female nude BALB/c mice (4–5 weeks) were maintained under a SPF environment, and the animal experiments were approved by the Institutional Animal Care and Use Committee of Nanjing Medical University (IACUC-2108034). Before the inoculation of mice, the H1299 cells transfected stably with LV-shCTR, LV-shp300, LV-shSTAT3 and LV-shMMP19 plus IL-17 (50 ng/mL) stimulation were selected with puromycin (2 μg/mL). Thereafter, the H1299 cells (1 × 10^6^) were respectively injected into the nude mouse via tail vein (100 μL/mouse, 5 mice/group). At 7 weeks, all mice were sacrificed, and the pulmonary metastatic nodules were counted and the tissue sections were observed by HE staining. Meantime, the expression of p300, p-STAT3, Ack-STAT3 and MMP19 in the lung metastatic tissues was examined by IP/IB.

### Statistical analysis

Statistical analysis was performed by GraphPad Prism software (San Diego, CA, USA). Two-tailed *t*-test was using to compare data between two groups, and multiple group comparison was analyzed by one-way ANOVA with Dunnett’s corrections. Data were shown as means ± SD from three independent experiments, and *p* < 0.05 was considered statistically significant. The correlations of IHC staining scores between the interest proteins were determined by computing Pearson’s correlation coefficient. Chi-square test was employed to evaluate the association between the protein expression and clinicopathological parameters of NSCLC patients.

## Results

### IL-17RA, p300, p-STAT3, Ack-STAT3 and MMP19 expression is raised in NSCLC tissues

Based on our reports that IL-17 was elevated in the sera of NSCLC patients [12] and many genes were overexpressed in fresh NSCLC tissues [8], we first selected and detected some genes associated with cancer metastasis according to previous sequencing [8], and relative literature [13–32]. The data showed that not only the mRNA of IL-17, IL-17RA, MMP1, MMP7, MMP9, MMP11, MMP13, MMP15, MMP19, KAT7, KAT8, PCAF and p300, especially IL-17, IL-17RA, MMP9, MMP19 and p300 (Suppl. Figs. S1A–S1C), but also the protein of MMP9, MMP19, p300 and IL-17RA (Suppl. Fig. S1D) and the phosphorylated or acetylated STAT1 and STAT3, namely p-STAT1, p-STAT3 or Ack-STAT1 and Ack-STAT3 (Suppl. Figs. S1E and S1F) were increased in 10 fresh NSCLC tissues. Because the levels of p300, p-STAT3, and MMP19 were higher than other molecules, thus we further examined IL-17RA, p300, p-STAT3 and MMP19 protein using NSCLC tissues arrays from 52 NSCLC patients (here, Ack-STAT3 did not detect owing to no commercial specific anti-Ack-STAT3). The IHC staining exhibited that these proteins were more than in adjacent tissues (Suppl. Figs. S2A and S2B) and had a positive relationship with each other (Suppl. Fig. S2C). Next, we analyzed the correlation between IL-17RA, p300, p-STAT3, or MMP19 expression and the clinic-pathological indicators of 52 NSCLC patients, and confirmed these proteins only were linked with lymph node metastasis and TNM stage (Suppl. Tables S4–S7), indicating the overexpression of these genes is associated with NSCLC progression.

### Cell migration and invasion or p300, MMP19, p-STAT3 and Ack-STAT3 expression are elevated or reduced in IL-17-stimulated or IL-17RA-silenced NSCLC cells

**Figure 1:**
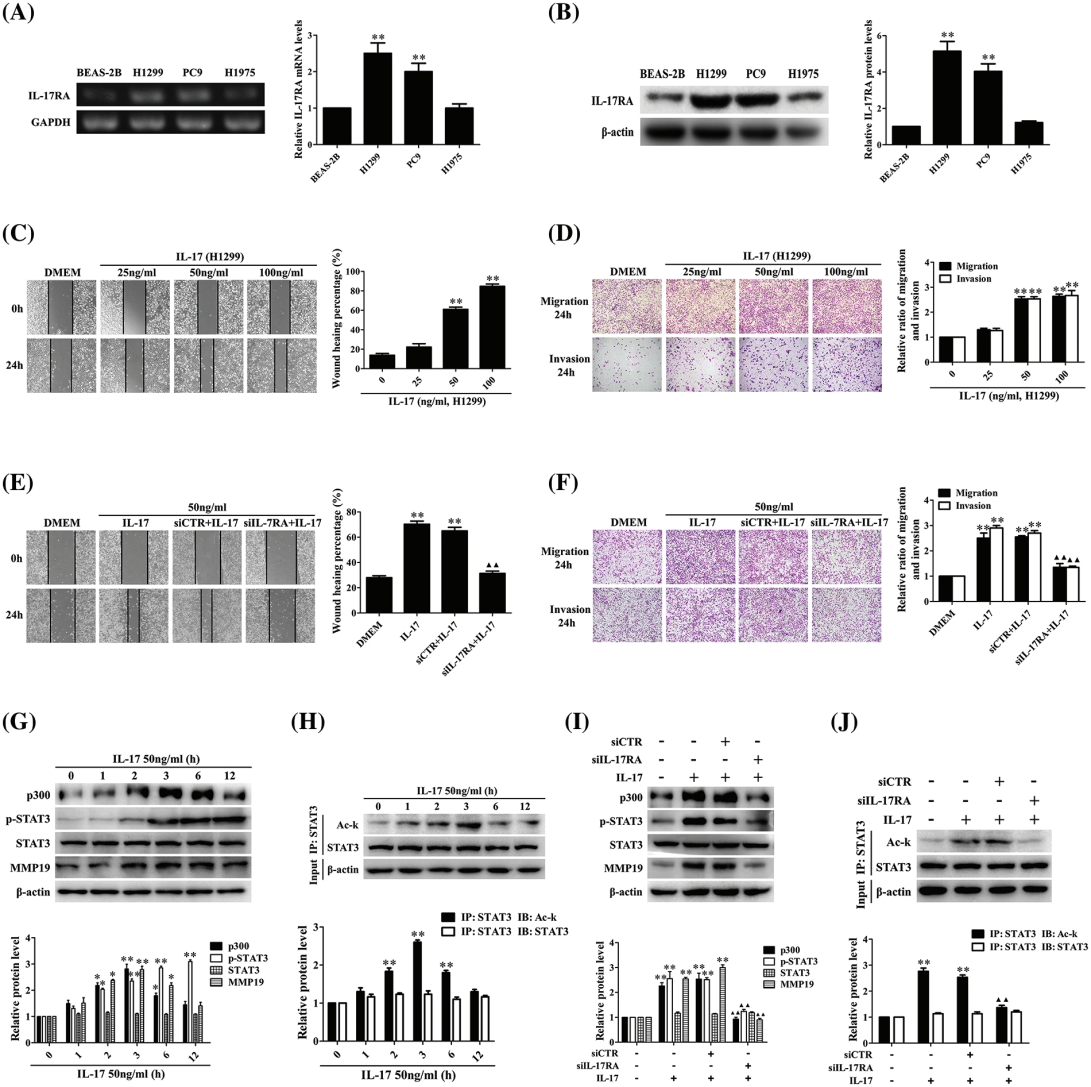
Expression of IL-17RA and effects of IL-17 stimulation or IL-17RA knockdown on H1299 cell migration and invasion as well as p300, p-STAT3, Ack-STAT3, and MMP19 expression. (A and B) IL-17RA mRNA (A) and protein (B) in the three NSCLC cell lines and normal BEAS-2B cell lines were examined by RT-PCR and IB (**p < 0.01 *vs*. BEAS-2B). (C and D) H1299 cell migration (C) and invasion (D) stimulated with various concentrations (ng/mL) of IL-17 for 24 h were detected by wound-healing and Transwell assays (**p < 0.01 *vs*. 0 ng/mL). (E and F) H1299 cells were treated with IL-17 (50 ng/mL) or transfected with siIL-17RA for 48 h followed by IL-17 (50 ng/mL) for 24 h, and the cell migration (E) and invasion (F) were examined by the same assays (**p < 0.01 *vs*. DMEM; ^▴▴^*p* < 0.01 *vs*. siCTR+IL-17). (G and H) Levels of p300, p-STAT3, STAT3, and MMP19 (G) or Ack-STAT3 (H) were measured using IB or IP/IB in H1299 cells exposed to 50 ng/mL IL-17 for different times (*p < 0.05, **p < 0.01 *vs*. 0 h). (I and J) Levels of p300, p-STAT3, STAT3, and MMP19 (I) or Ack-STAT3 (J) in H1299 cells stimulated with IL-17 (50 ng/mL) or transfected with siIL-17RA plus IL-17 (50 ng/mL) for 3 h were assessed using IB or IP/IB (**p < 0.01 *vs*. DMEM; ^▴▴^*p* < 0.01 *vs*. siCTR+IL-17). Representative pictures are shown. Data from three independent experiments are expressed as means ± SD and analyzed by one-way ANOVA (A–J).

Numerous researches indicate that IL-17 up-regulation in NSCIC tissues contributes to NSCLC metastasis [10,13,21], but the mechanism of IL-17-induced NSCLC metastasis has not been fully known. To solve this problem, we first detected IL-17RA in three NSCLC cell lines and normal bronchial epithelium cell line BEAS-2B, and meanwhile, we also examined the expression levels of p300, p-STAT3, STAT3, and MMP19 in these cell lines. The results showed that mRNA and protein of IL-17RA were highly expressed in H1299 and PC9 cells (Figs. 1A and 1B), and the protein of p300, p-STAT3, STAT3, and MMP19 could be seen and the expression levels of these proteins were similar in the four cell lines (Suppl. Fig. S3A). Next, we exploited H1299 and PC9 cells with IL-17 to observe cell migration and invasion and proved that the cell behavior was markedly enhanced at 50 and 100 ng/mL IL-17 (Figs. 1C and 1D, Suppl. Figs. S3B and S3C). Because the changes of cell migration and invasion in the two cells treated with 50 or 100 ng/mL IL-17 had no statistical significance (*p* > 0.05), then we further detected the cell migration in H1299 and PC9 cells stimulated with 50 ng/mL IL-17 and confirmed that the cell migration was augmented at 24 h, particularly markedly in H1299 cells (Suppl. Figs. S3E and S3F). Moreover, we transfected siIL-17RA into H1299 cells to silence the IL-17RA gene (Suppl. Fig. S3D) followed by 50 ng/mL IL-17 stimulation for 24 h and found that H1299 cell migration and invasion did not enhance (Figs. 1E and 1F), implying IL-17-induced these changes truly depend on IL-17-IL-17RA combination.

According to our previous sequencing data and related documents [8,13–32], we chose and screened the mRNA of some transcriptional factors, HATs, and MMPs in H1299 cells exposed to 50 ng/mL IL-17. After demonstrating that the mRNA levels of p300, MMP2, MMP9, MMP13, and MMP19 were increased at 2 h, and peaked at 3 h, especially p300 and MMP19 (Suppl. Figs. S4A–S4C), we further detected the protein of p300, MMP19, STAT3, p-STAT3, and Ack-STAT3 in response to IL-17 (50 ng/mL). The results displayed that p300 and MMP19 mRNA (Suppl. Fig. S4D) and p300, MMP19, Ack-STAT3 protein (Figs. 1G and 1H) were up-regulated at 2 h, and peaked at 3 h, and p-STAT3 also elevated at 3 h, continued to increase for 12 h (Fig. 1G). Contrarily, these indicators in the cells transfected with siIL-17RA plus IL-17 stimulation were not elevated (Suppl. Fig. S4E, Figs. 1I and 1J), suggesting that the expression phases of these genes elevated by IL-17 binding to IL-17RA are synchronous.

### p300 and STAT3 down-regulation can inhibit IL-17-induced H1299 cell migration, invasion and MMP19 expression

**Figure 2:**
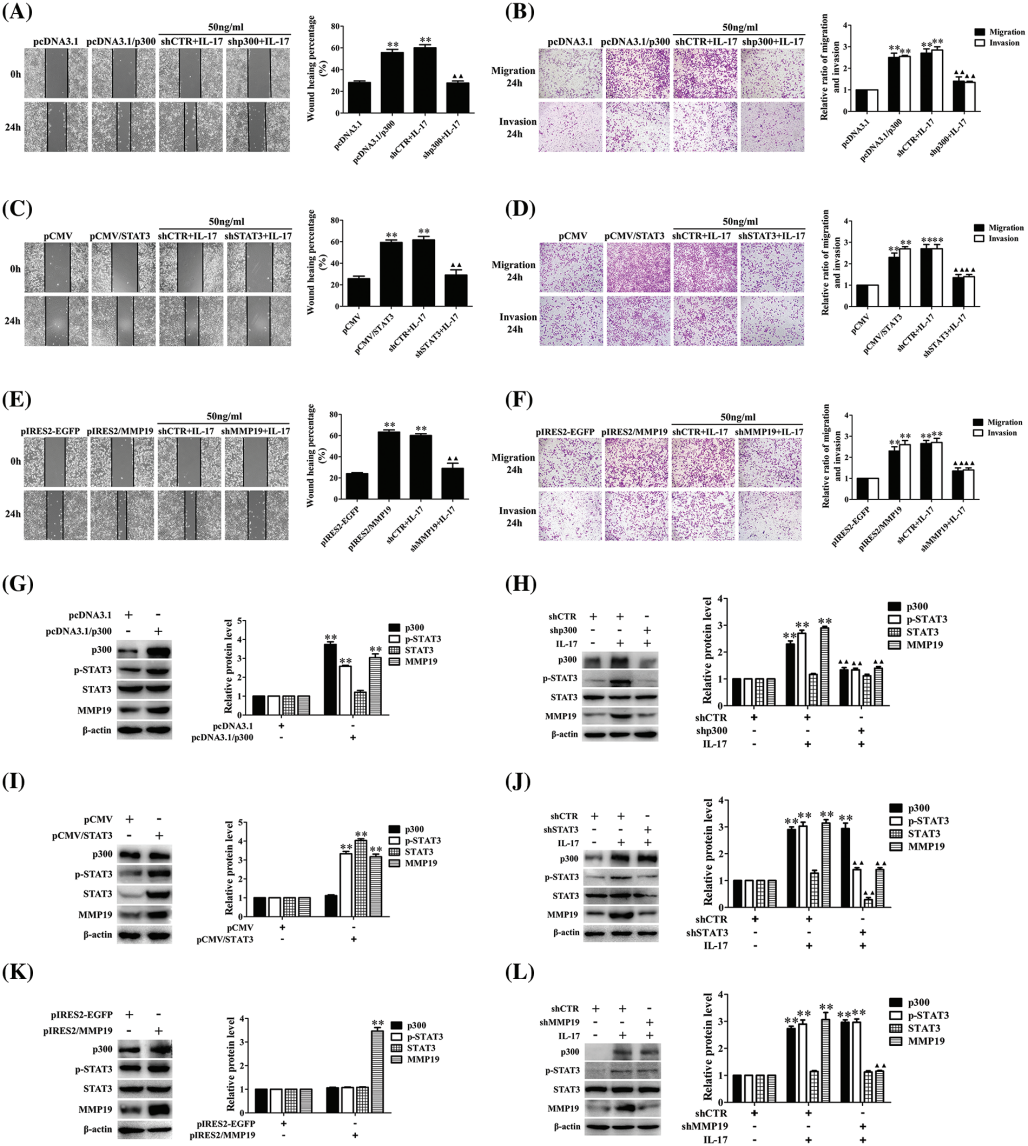
Changes of cell migration or invasion and MMP19 expression in H1299 cells after p300, STAT3, and MMP19 overexpression or knockdown. (A–F) H1299 cell migration and invasion in the cells transfected with pcDNA3.1/p300, pCMV/STAT3, and pIRES2/MMP19, or transfected with shp300, shSTAT3 and shMMP19 followed by IL-17 (50 ng/mL) stimulation for 24 h were observed by wound-healing and Transwell assays. (G–L) Expression of p300, p-STAT3, STAT3, and MMP19 protein in the cells transfected with the above-mentioned overexpression (G, I, K) or shRNA (H, J, L) plasmids plus 50 ng/mL IL-17 for 3 h was detected using IB. Representative pictures are exhibited. All data from three independent experiments are displayed as means ± SD and analyzed by one-way ANOVA (A–F, H, J, L) or *t*-test (G, I, K), **p < 0.01 *vs*. pcDNA3.1, pCMV, pIRES2/EGFP, or shCTR; ^▴▴^*p* < 0.01 *vs*. shCTR+IL-17.

Because p300, STAT3, and MMP19 expression in H1299 cells upon IL-17 was earlier than the cell mobility, the role of these three molecules in cell migration and invasion needs to be clarified. Therefore, we respectively transfected the overexpressing plasmids of p300, STAT3, and MMP19 (i.e., pcDNA3.1/p300, pCMV/STAT3, pIRES2/MMP19) or the interfering plasmids (i.e., shp300, shSTAT3, shMMP19) into H1299 cells after ensuring that these plasmids worked well (Suppl. Figs. S5A–S5G). We observed that the p300, STAT3, and MMP19 overexpression accelerated the cell migration and invasion. On the other hand, when H1299 cells were transfected with shp300-2, shSTAT3-2, or shMMP19-4 (Suppl. Figs. S5E–S5G), the IL-17-induced cell migration and invasion were inhibited (Figs. 2A–2F). Besides, we also found that p300 and STAT3 gene overexpression or knockdown could respectively increase or decrease p-STAT3 and MMP19 expression (Figs. 2G–2J), but MMP19 overexpression or knockdown did not alter p300 and STAT3 levels (Fig. 2K and 2L). These data indicate that MMP19 is a downstream molecule of p300 and STAT3 in IL-17-stimulated H1299 cells, and p300 or STAT3 up-regulation can promote MMP19 induction.

### IL-17 stimulation or p300 and STAT3 overexpression can enhance MMP19 promoter activity

Although we have exhibited that p300 and STAT3 can regulate MMP19 expression, whether p300 or STAT3 affects MMP19 gene initiation is still unrevealed. Thus, we constructed the plasmids of the pGL3-MM19-FL (−1163 to +313 nt) promoter (Suppl. Figs. S6A and S6B). Then, H1299 cells were stimulated with 50 ng/mL IL-17 or co-transfected pGL3-MM19-FL together with pcDNA3.1/p300, pCMV/STAT3 plasmids, or siIL-17RA, shp300, shSTAT3 plasmids followed by IL-17, respectively. The results displayed that IL-17 stimulation, or p300, STAT3 overexpression elevated MMP19-FL promoter activity, while IL-17RA, p300, or STAT3 gene silence reduced IL-17-elevated MMP19 promoter activity (Suppl. Figs. S6C–S6E), suggesting that IL-17 treatment and p300 or STAT3 overexpression can activate MMP19 gene.

### STAT3 and p300 can bind to the same region of the MMP19 gene promoter in IL-17-stimulated H1299 cells

**Figure 3:**
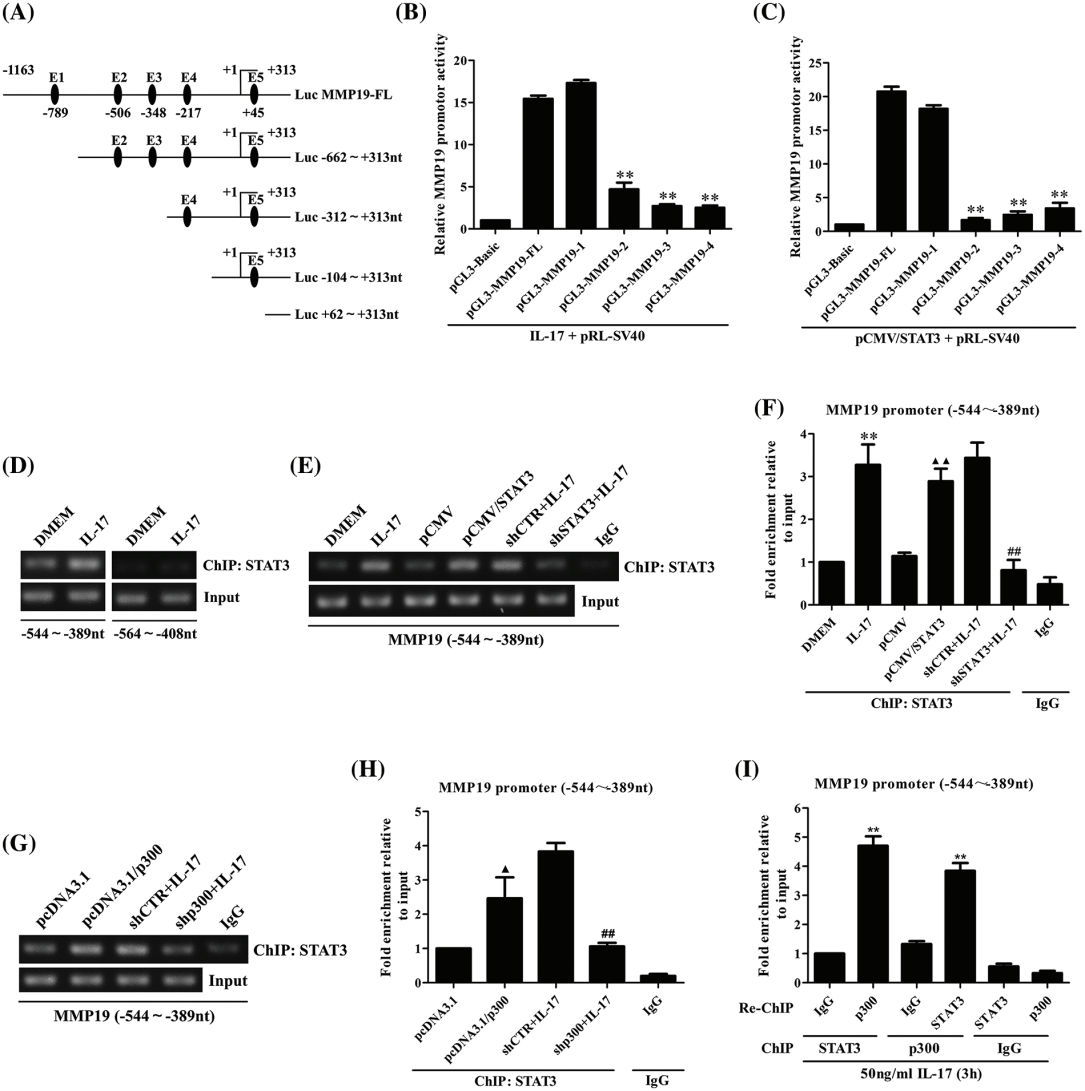
Identification of STAT3 binding to MMP19 promoter, and the effect of STAT3 or p300 overexpression on the binding of STAT3 to MMP19 promoter. (A) Full-length (FL) and truncated fragments of the MMP19 promoter were shown. (B) H1299 cells were transfected with MMP19 FL and truncated promoter respectively, and the cells were stimulated with IL-17 (50 ng/mL) for 3 h, MMP19 promoter activity was evaluated by luciferase reporter assay (**p < 0.01 *vs*. pGL3-MMP19-FL+IL-17). (C) MMP19 promoter plasmids above-mentioned were co-transfected with pCMV/STAT3, and MMP19 promoter activity was detected (**p < 0.01 *vs*. pGL3-MMP19-FL+pCMV/STAT3). (D) H1299 cells were stimulated by IL-17 (50 ng/mL) for 3 h, and ChIP assay was done with STAT3 Ab, and PCR was employed to measure the corresponding region of the MMP19 promoter. (E–H) H1299 cells were treated with 50 ng/mL IL-17 or transfected by STAT3 or p300 overexpression plasmids for 48 h or interferon plasmids for 48 h plus the same dose of IL-17 for 3 h. ChIP was carried out. The −544 to −389 nt fragment of the MMP19 promoter was detected by RT-PCR (E, G) and real-time PCR (F, H). **p < 0.01 *vs*. DMEM; ^▴^*p* < 0.05 *vs*. pcDNA3.1; ^▴▴^*p* < 0.01 *vs*. pCMV; ^##^*p* < 0.01 *vs*. shCTR+IL-17. (I) H1299 cells were stimulated with 50 ng/mL IL-17 for 3 h. The anti-STAT3 Ab was used for ChIP, and anti-p300 Ab was used for re-ChIP, or vice versa. The MMP19 promoter fragment described previously was examined by real-time PCR (**p < 0.01 *vs*. IgG). Data from triplicate experiments are expressed as means ± SD and analyzed by one-way ANOVA (B, C, F, H, I).

Given that STAT3 and p300 can up-regulate MM19 promoter activity, the site of STAT3 or p300 binding to MMP19 promoter should be searched. Thereupon, we predicted STAT3-response elements on MMP19 promoter using JASPAR software (Fig. 3A) and then constructed truncated MMP19 promoter plasmids (truncate 1: −662 to +313 nt; truncate 2: −312 to +313 nt; truncate 3: −104 to +313 nt; and truncate 4: +62 to +313 nt). Subsequently, we transfected MMP19-FL or different truncated promoter plasmids plus 50 ng/mL IL-17 or co-transfected above-mentioned plasmids with pCMV/STAT3 into H1299 cells, and found that MMP19 promoter activity of truncate 2, 3 and 4 was down-regulated both in IL-17-treated and STAT3-overexpressed cells compared with MMP19-FL (Figs. 3B and 3C), hinting that the STAT3-binding elements may locate in −662 to −312 nt region of MMP19 promoter.

Because the −662 to −312 nt of the MMP19 promoter has STAT3 binding elements predicted by JASPAR, we then designed the primers that contained the two elements (−544 to −389 nt and −564 to +313 nt) to do ChIP assay. The results exhibited that IL-17-activated STAT3 could bind to the −544 to −389 nt region of the MMP19 promoter (Fig. 3D), and STAT3 gene overexpression or knockdown could up-regulate or down-regulate its binding to this site of MMP19 promoter (Figs. 3E and 3F). These findings reveal that STAT3 binding element locates within −544 to −389 nt of the MMP19 promoter.

Moreover, ChIP and re-ChIP manifested that p300 gene overexpression or knockdown also increased or decreased its binding to −544 to −389 nt of the MMP19 promoter (Figs. 3G and 3H). Meanwhile, p300 and STAT3 could form a complex binding to the same site of MMP19 promoter in a STAT3-dependent way (Fig. 3I), implying that p300 also promotes IL-17-induced MMP19 gene transcription.

### IL-17 stimulation, p300 overexpression or knockdown and activity inhibition can regulate p300-STAT3 combination, STAT3 acetylation and phosphorylation

**Figure 4:**
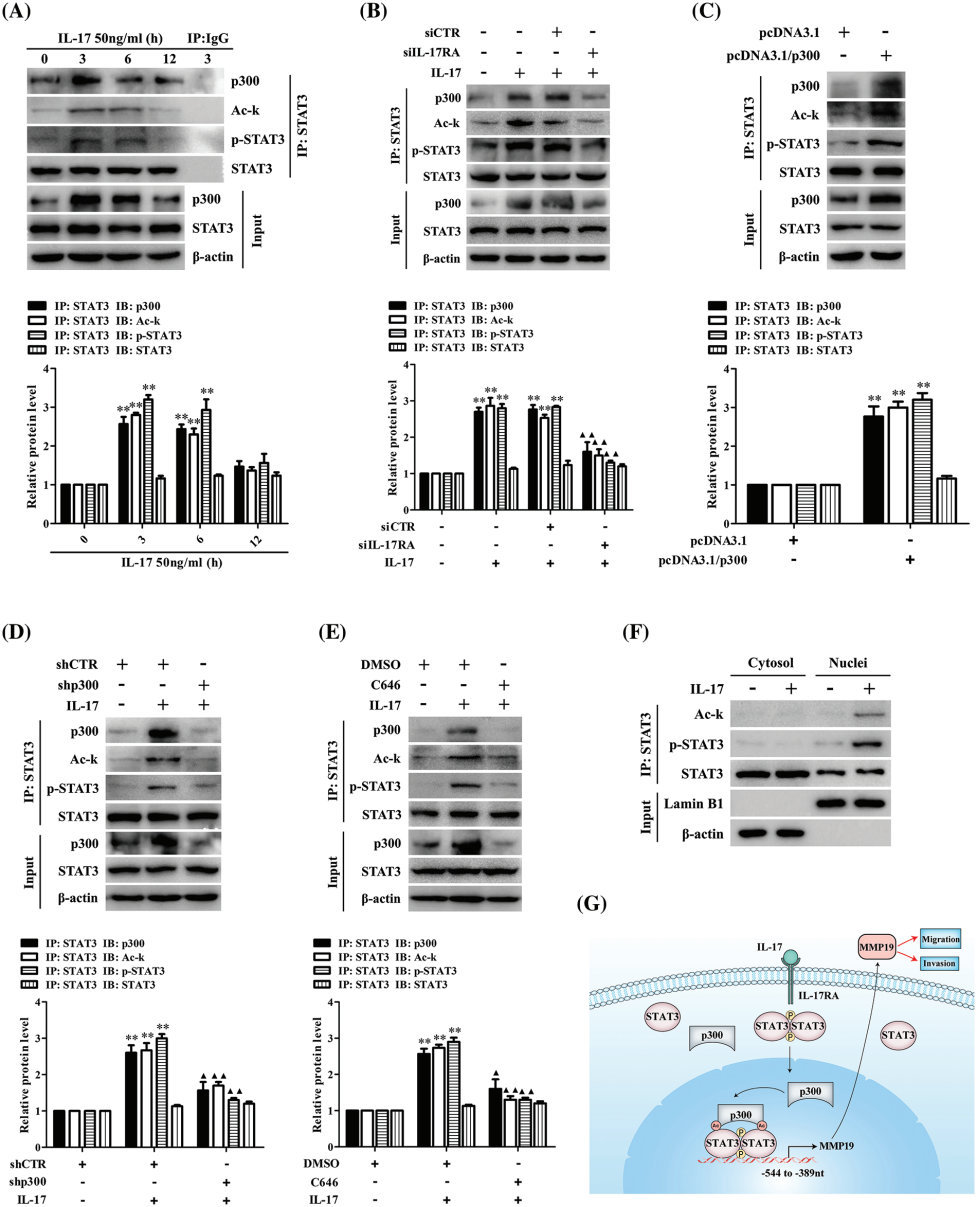
STAT3 binding to p300 as well as STAT3 acetylation and phosphorylation in H1299 cells upon IL-17 stimulation, p300 gene knockdown or activity inhibition, and the putative scheme for IL-17-induced metastasis mechanism. (A) H1299 cells were exposed to 50 ng/mL IL-17 for various time, the levels of STAT3-p300 combination, Ack-STAT3 and p-STAT3 were measured using IP/IB (**p < 0.01 *vs*. 0 h). (B) Cells were transfected with siIL-17RA for 48 h plus 50 ng/mL IL-17 for 3 h. The STAT3 binding to p300, Ack-STAT3 and p-STAT3 were detected by IP/IB (**p < 0.01 *vs*. DMEM; ^▴^*p* < 0.05, ^▴▴^*p* < 0.01 *vs*. siCTR+IL-17). (C and D) Cells were transfected with pcDNA3.1/p300 (C) or shp300 (D) plasmids for 48 h plus 50 ng/mL IL-17 for 3 h, the STAT3-p300 combination, Ack-STAT3 and p-STAT3 were identified by IP/IB (**p < 0.01 *vs*. pcDNA3.1 or shCTR; ^▴^*p* < 0.05, ^▴▴^*p* < 0.01 *vs*. shCTR+IL-17). (E) Cells were treated with C646 for 3 h followed by 50 ng/mL IL-17 for 3 h, and the p300 binding to STAT3, Ack-STAT3 and p-STAT3 were assessed by IP/IB (**p < 0.01 *vs*. DMSO; ^▴^*p* < 0.05, ^▴▴^*p* < 0.01 *vs*. DMSO+IL-17). (F) Cells were treated with 50 ng/mL IL-17 for 3 h, and the distribution of Ack-STAT3 and p-STAT3 protein was observed using IP/IB. Data from triplicate experiments are shown as means ± SD and analyzed by one-way ANOVA (A, B, D, E) or *t*-test (C). (G) The putative scheme for IL-17-induced NSCLC metastasis mechanism. The p300 and p-STAT3 are up-regulated by IL-17/IL-17RA, and then they are translocated into H1299 cell nucleus binding to the same region (−544 to −389 nt) of MMP19 promoter. Meantime, p300 acetylates STAT3-K631 enhancing STAT3 binding and p-STAT3 level, while p-STAT3 also elevates Ack-STAT3 level, and Ack-STAT3 and p-STAT3 interaction promotes MMP19 gene transcription and expression promoting NSCLC cell migration and invasion.

It is accepted that p300 can modify the lysine of histones and transcription factors [29–31], but whether p300 can acetylate STAT3 promoting MMP19 gene transcription in IL-17-treated H1299 cells remains unclear. Hence, we did IP/IB tests and found a robust interaction between p300 and STAT3 in H1299 cells under IL-17 exposure. Meantime, STAT3 acetylation and its Y705-phosphorylation (i.e., p-STAT3) were also enhanced (Fig. 4A), but IL-17RA-silenced cells followed by IL-17 stimulation obtained the opposite effect (Fig. 4B). Furthermore, to investigate that p300 can acetylate STAT3 and affect its p-STAT3, we transfected pcDNA3.1/p300 into H1299 cells and confirmed that the levels of p300 binding to STAT3, Ack-STAT3 and p-STAT3 were all increased (Fig. 4C). However, the same results did not appear in H1299 cells with shp300 or 10 μmol/L C646 (p300 inhibitor) plus 50 ng/mL IL-17 (Figs. 4D and 4E). Besides, we proved that the distribution of Ack-STAT3 and p-STAT3 in H1299 cells exposed to IL-17 was mainly located in the cell nucleus (Fig. 4F). These findings support that IL-17-up-regulated p300 can acetylate STAT3 and impact its phosphorylation via p300 binding to STAT3.

### STAT3 acetylation site mediated by p300 is K631 in H1299 cells in response to IL-17

**Figure 5:**
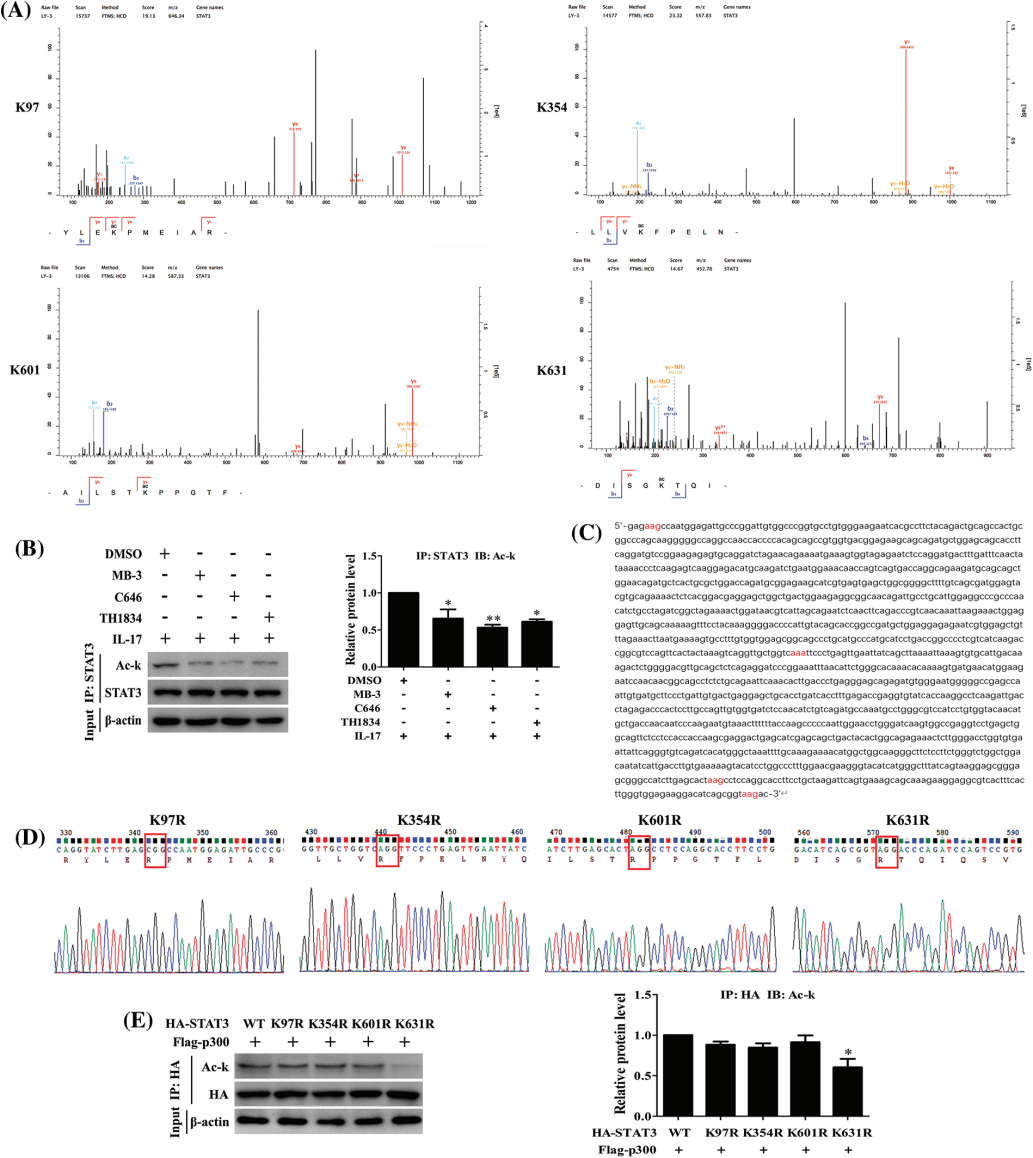
IL-17-induced lysine acetylation of STAT3 mediated by p300. (A) H1299 cells were treated with 50 ng/mL IL-17 for 3 h. IP assay with STAT3 Ab was performed, and the purified products were subjected to mass spectrometry to find the sites of STAT3 lysine acetylation induced by IL-17. (B) The cells were treated with the inhibitors of three acetyltransferases, i.e., MB-3 (GCN5 inhibitor, 5 μmol/L), C646 (p300 inhibitor, 10 μmol/L) and TH1834 (Tip60 inhibitor, 100 μmol/L) for 3 h followed by 50 ng/mL IL-17 stimulation for 3 h, and the level of STAT3 acetylation was detected by IP and IB (*p < 0.05, **p < 0.01 *vs*. DMSO+IL-17). (C) Parts of the CDS region of STAT3, the codons of STAT3 acetylation sites (K97, K354, K601 and K631) were marked red. (D) The four non-acetylated STAT3 mutant plasmids (K97R, K354R, K601R and K631R) were constructed, and the parts of sequencing results were shown. (E) H1299 cells were transfected with the above-mentioned mutant plasmids, and STAT3 acetylation was measured by IP and IB (*p < 0.05 *vs*. HA-STAT3 wild-type (WT) + Flag-p300). All data from triplicate experiments are presented as means ± SD, and analyzed by one-way ANOVA (B, E).

Since we have found that IL-17-induced STAT3 acetylation is p300-dependent (Fig. 5B), we thus continued to search which lysine (K) of STAT3 could be acetylated. The mass spectrometry (MS) detection uncovered that STAT3-K97, -K354, -K601 and -K631 were all acetylated in H1299 cells upon 50 ng/mL IL-17 (Fig. 5A). Next, to further confirm the effective site of p300-acetylated STAT3, we mutated the four lysine of STAT3 by altering K to non-acetylatable arginine (R) (Figs. 5C and 5D), and then co-transfected these mutants into H1299 cells with HA-p300. The experiments showed that STAT3 acetylation level was markedly reduced in the cells con-transfected STAT3-K631R with HA-p300 (Fig. 5E), implicating that the effective site of p300-mediated STAT3 acetylation is K631.

### STAT3 acetylation can up-regulate its binding to MMP19 promoter or MMP19 promoter activity, p-STAT3, MMP19 expression, cell migration and invasion

**Figure 6:**
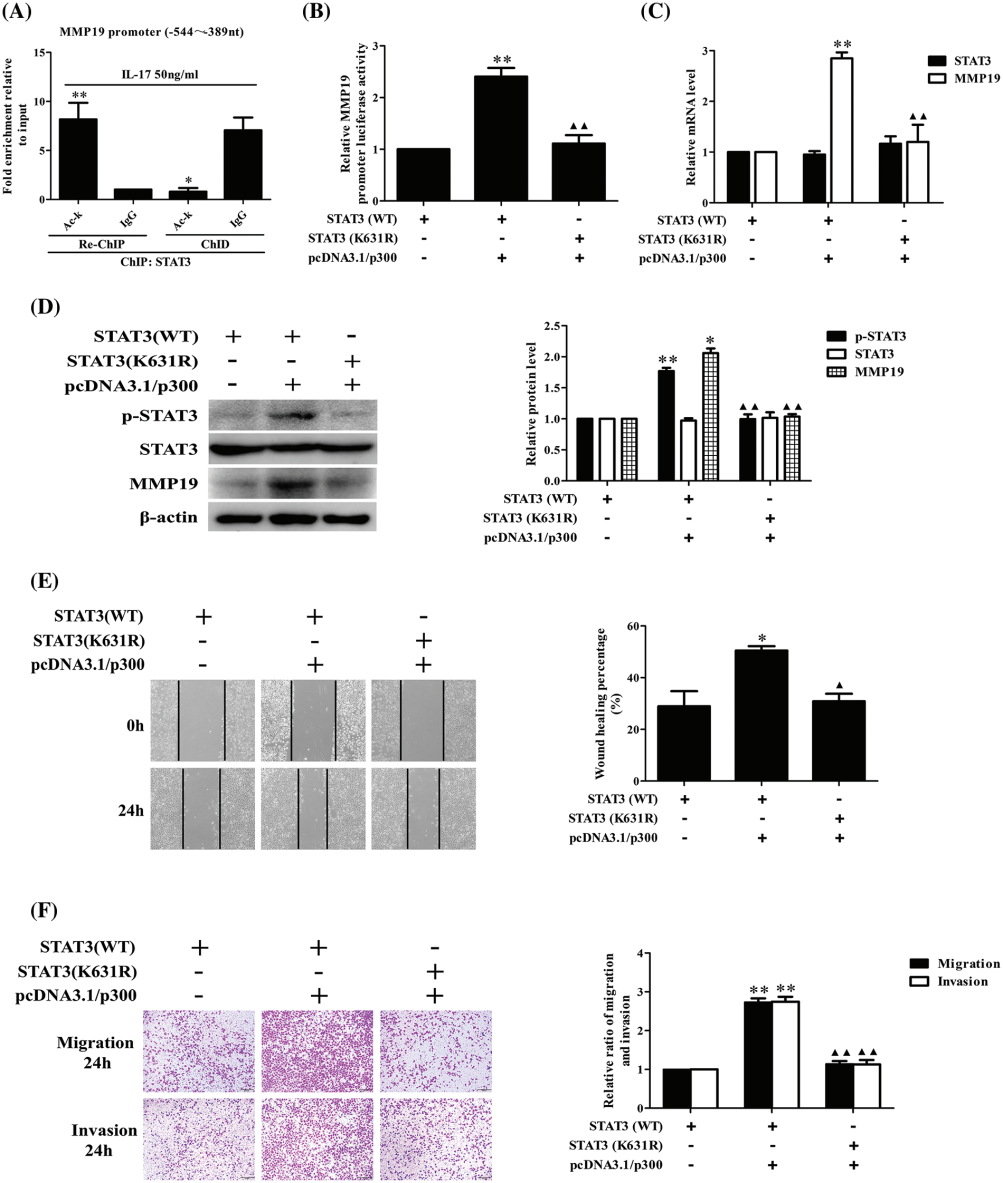
Effects of STAT3 acetylation on STAT3 phosphorylation, MMP19 gene transcription and expression as well as cell migration and invasion. (A) H1299 cells were stimulated by 50 ng/mL IL-17 for 3 h, and ChIP was performed with anti-STAT3, and anti-Ack Ab was used for the following re-ChIP. The MMP19 promoter fragment in the sediment (re-ChIP) and supernatant (ChID) was detected by real-time-PCR (*p < 0.05, **p < 0.01 *vs*. IgG). (B–D) STAT3 (WT) and STAT3-K631 mutant (K631R) plasmids were co-transfected with pcDNA3.1/p300 into H1299 cells for 48 h, and MMP19 promoter activity (B), mRNA (C), as well as MMP19 protein and p-STAT3 (D) were assessed by luciferase reporter, real-time PCR and IB (*p < 0.05, **p < 0.01 *vs*. STAT3(WT), ^▴▴^*p* < 0.01 *vs*. STAT3(WT) + pcDNA3.1/p300). (E and F) Cells were transfected with above-mentioned plasmids for 48 h, the cell migration (E) and invasion (F) were examined by wound-healing and Transwell assay (*p < 0.05, **p < 0.01 *vs*. STAT3(WT); ^▴^*p* < 0.05, ^▴▴^*p* < 0.01 *vs*. STAT3(WT) + pcDNA3.1/p300). Representative pictures and data were obtained from triplicate experiments. Data (means ± SD) are analyzed by one-way ANOVA (A–F).

Because p300 expression can increase STAT3 binding to MMP19 promoter, we assume that p300-acetylated STAT3 can augment this binding capacity leading to cell behavior changes. To give weight to this speculation, we performed ChIP, re-ChIP and ChID assays, and found that acetylated-STAT3 binding to −544 to −389 nt of MMP19 promoter in IL-17-stimulated H1299 cells was greatly enhanced (Fig. 6A). Additionally, the levels of MMP19 promoter activity, mRNA, protein and p-STAT3 expression (Figs. 6B–6D) as well as cell migration and invasion (Figs. 6E and 6F) were also increased or decreased in the cells co-transfected with STAT3 (WT), p300 or STAT3 (K631R) and p300 plasmids. These results disclose that p300-mediated STAT3-K631 acetylation can elevate its binding capability to specific sites of MMP19 promoter, MMP19 promoter activity, p-STAT3 and MMP19 expression as well as cell migration and invasion.

### STAT3-Y705 phosphorylation inhibition or mutation can affect IL-17-induced STAT3 acetylation, MMP19 expression, cell migration and invasion

**Figure 7:**
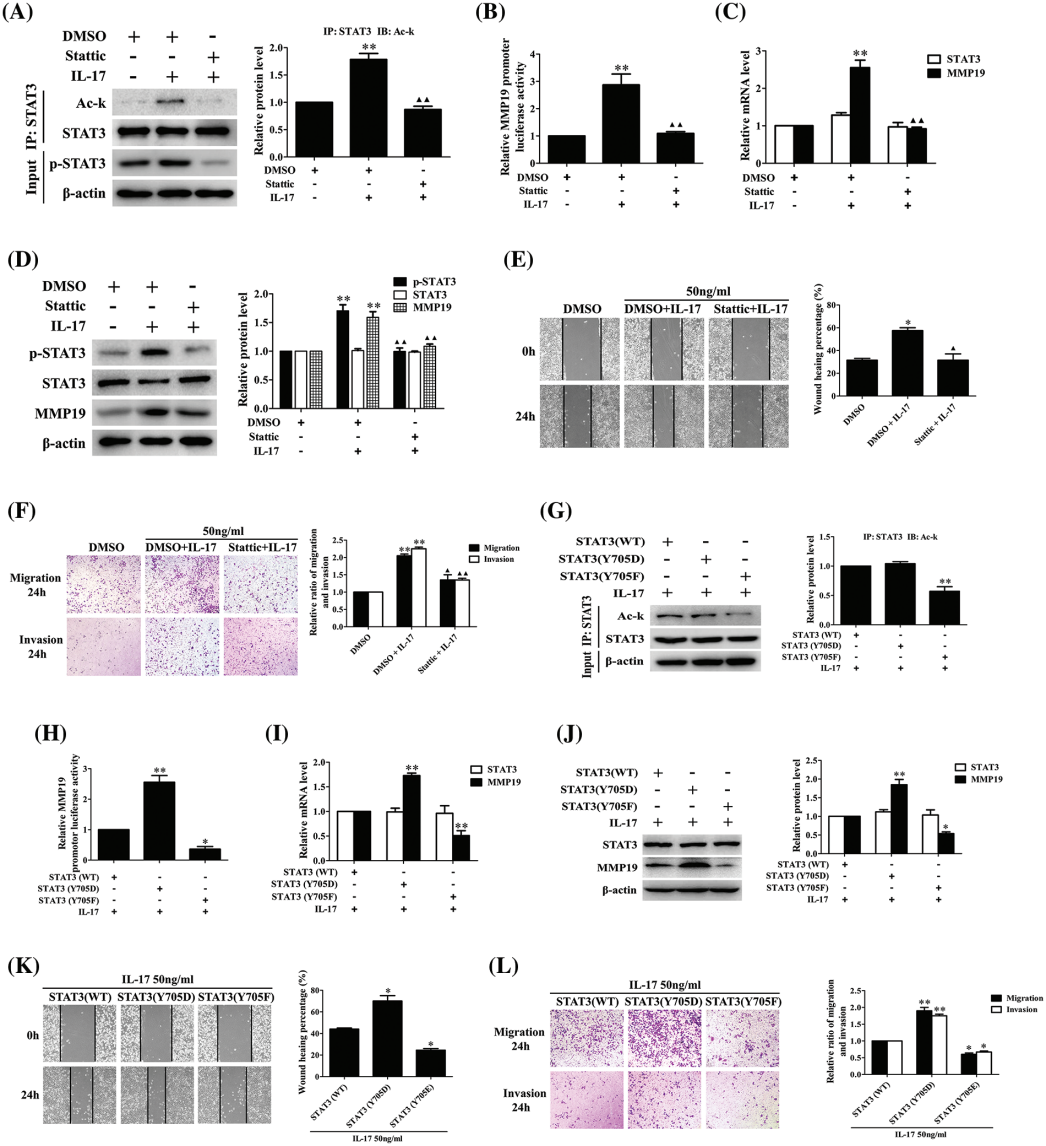
STAT3 phosphorylation, acetylation, MMP19 gene transcription and expression as well as cell migration and invasion in H1299 cells treated with Stattic or p-STAT3-Y705 activated or inactivated mutants plus IL-17 stimulation. (A–D) Cells were pre-treated with the 10 μmol/L Stattic for 3 h and followed by 50 ng/mL IL-17 for 3 h. Then, the levels of STAT3 acetylation (A), MMP19 promoter activity (B), MMP19 mRNA (C) and protein (D) were assessed by luciferase reporter, real-time PCR or IB (**p < 0.01 *vs*. DMSO; ^▴▴^*p* < 0.01 *vs*. DMSO+IL-17). (E and F) Cells were pre-treated with Stattic (10 μmol/L) for 3 h plus IL-17 (50 ng/mL) for 24 h, the cell migration (E) and invasion (F) were evaluated by wound-healing and Transwell assay (*p < 0.05, **p < 0.01 *vs*. DMSO; ^▴^*p* < 0.05, ^▴▴^*p* < 0.01 *vs*. DMSO+IL-17). (G) STAT3 (WT) and STAT3 (Y705D) or STAT3 (Y705F) plasmids were transfected into H1299 cells for 48 h plus 50 ng/mL IL-17 for 3 h, and Ack-STAT3 was detected using IP/IB (**p < 0.01 *vs*. STAT3 (WT)). (H–J) Cells were transfected with STAT3 (WT), STAT3 (Y705D) or STAT3 (Y705F) for 48 h followed by 50 ng/mL IL-17 for 3 h. MMP19 promoter activity (H), MMP19 mRNA (I) and protein (J) were measured by luciferase reporter, real-time PCR or IB, *p < 0.05, **p < 0.01 *vs*. STAT3 (WT). (K and L) Cells were transfected with STAT3 (WT), STAT3 (Y705D) or STAT3 (Y705F) for 48 h plus 50 ng/mL IL-17 for 24 h, and the cell migration (K) and invasion (L) were observed by wound-healing and Transwell assay, *p < 0.05, **p < 0.01 *vs*. STAT3 (WT). Representative pictures are exhibited and the data (means ± SD) from triplicate experiments were analyzed by one-way ANOVA (A–L).

Our foregoing tests have demonstrated that p300-dependent STAT3 acetylation can boost its Y705-phosphorylation (p-STAT3), whether p-STAT3 can influence its acetylation (Ack-STAT3) or cell migration and invasion is worth exploring. Our data displayed that the Ack-STAT3 and p-STAT3 level, MMP19 promoter activity, mRNA and protein expression (Figs. 7A–7D) as well as cell migration and invasion (Figs. 7E and 7F) were all down-regulated in H1299 cells pre-treated with 10 μmol/L Stattic (p-STAT3-Y705 inhibitor) [23] plus IL-17 stimulation. Moreover, we respectively transfected the plasmids of phosphorylation-activated-STAT3(Y705D) or -inactived-STAT3(Y705F) followed by IL-17, and found that despite STAT3(Y705F) did not facilitate STAT3 acetylation (Fig. 7G), other above-described parameters could accordingly up-regulate or down-regulate (Figs. 7H–7L), indicating that the interplay between STAT3 acetylation and its phosphorylation can synergistically promote MMP19 induction, cell migration and invasion in IL-17-exposed to H1299 cells.

### Lung metastatic nodules and relative protein expression are alleviated in the metastatic model of BALB/c nude mice

**Figure 8:**
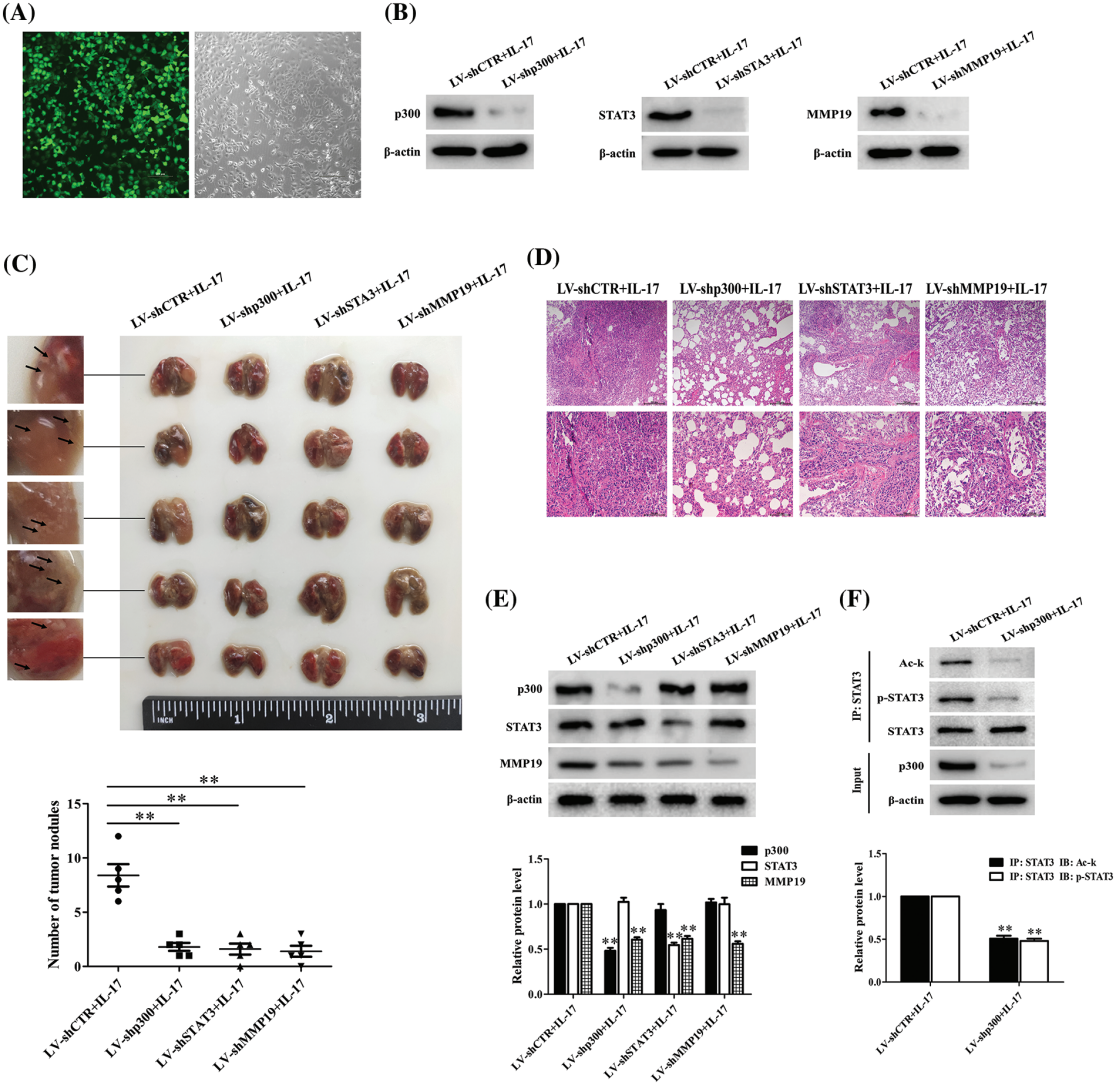
Effects of lung metastasis and relative protein expression in BALB/c nude mice inoculated with H1299 cells by silencing p300, STAT3 or MMP19 gene plus 50 ng/mL IL-17 stimulation. (A) H1299 cells were infected with LV-shCTR, LV-shp300, LV-shSTAT3 or LV-shMMP19, and the infected efficiency was assessed by GFP (scale bar: 200 μm). (B) H1299 cells infected with above-mentioned LV-shRNA were selected with 2 μg/mL puromycin and then stimulated with 50 ng/mL IL-17 for 3 h. The corresponding protein expression was examined using IB to verify the gene knockdown. (C) BALB/c nude mice (n = 5/group) were intravenously injected H1299 cells previously described, and at 7 weeks after inoculation, the mice were sacrificed and lung tissues were collected to count the number of lung metastatic nodules (**p < 0.01 *vs*. LV-shCTR+IL-17). (D) Morphological sections of lung metastatic nodules were observed by HE staining (scale bar: top, 200 μm or bottom, 100 μm). (E) The p300, STAT3 and MMP19 expression of the lung metastatic tissues in different groups were detected by IB (**p < 0.01 *vs*. LV-shCTR+IL-17). (F) STAT3 acetylation and phosphorylation levels in the corresponding groups were measured by IP/IB (**p < 0.01 *vs*. LV-shCTR+IL-17). Representative pictures are shown, and data from each group of mice are expressed as means ± SD and analyzed by one-way ANOVA (C, E) or *t*-test (F).

Given that we have discovered the pro-metastasis role of p300, STAT3 and MMP19 *in vitro*, we wonder these protein functions *in vivo*. Thus, we infected H1299 cells with LV-packaging shCTR, shp300, shSTAT3 or shMMP19 plasmids and followed by 50 ng/mL IL-17 stimulation. After the corresponding gene knockdown was verified (Figs. 8A and 8B), we injected intravenously these cells treated with the different LV-shRNA into BALB/c nude mice, respectively. After the inoculation for 7 weeks, we sacrificed all mice and examined the relative parameters. The results exhibited that the lung metastatic foci number and pathological change in the mice of LV-shp300+IL-17, LV-shSTAT3+IL-17 or LV-shMMP19+IL-17 group were significantly less or lighter than those in LV-shCTR+IL-17 group (Figs. 8C and 8D). Meantime, MMP19 expression, or p-STAT3 and Ack-STAT3 of lung metastatic cancer tissues in the corresponding groups were also declined (Figs. 8E and 8F). These results support that p300, STAT3 and MMP19 expression can indeed accelerate NSCLC cell metastasis.

## Discussion

NSCLC is an inflammation-related carcinoma [8,12,33], and pro-inflammatory IL-17 expression in NSCLC tissues can promote cancer metastasis [10,13,34]. Although IL-17-induced NSCLC metastasis has been confirmed, the underlying mechanism of NSCLC cell metastasis is still unilluminated. In the study, we selected and measured the expression of the genes which were reported to correlate with cancer metastasis according to the information of NSCLC tissue sequencing [8] and relevant literature [13–32], and found that the expression levels of IL-17RA, p300, p-STAT3, Ack-STAT3 and MMP19 were significantly higher than other gene in NSCLC samples, suggesting that these proteins may play some roles in NSCLC metastasis.

Generally, the cell reaction stimulated by cytokine, e.g., IL-17 needs binding to its receptor on the cells [13,35]. Hence, we first examined IL-17RA expression in three NSCLC cell lines and normal bronchial epithelium cell line (BEAS-2B) and proved that IL-17RA levels in H1299 and PC9 cells were more than BEAS-2B cells. Next, we observed the cell migration and invasion in H1299 and PC9 cells stimulated by IL-17 or transfected siIL-17RA plus IL-17, and demonstrated that IL-17 stimulation enhanced the cell migration and invasion, while IL-17RA gene knockdown inhibited these changes induced by IL-17, particularly in H1299 cells. Thereupon, we further used H1299 cells to measure the levels of p300, STAT3, p-STAT3, Ack-STAT3, and MMP19 expression in response to IL-17 or IL-17RA knockdown plus IL-17, and data showed that the changes of above-mentioned parameters were the similar to those of H1299 cell migration and invasion described previously. These results implicate that the cell reactions caused by IL-17 are dependent on IL-17 binding to IL-17RA in the cells.

Emerging studies have discovered that STAT3, p300 and MMP19 are overexpressed in breast cancer [36], esophageal cancer [37], colorectal cancer (CRC) [38] or NSCLC [15] and so on [16,31], promoting cancer cell growth or metastasis. Therefore, we explored the role of p300, STAT3 and MMP19 in IL-17-induced H1299 cell migration and invasion as well as their relationship, and affirmed that the overexpression of these genes could augment the cell migration and invasion, but the knockdown of these genes had contrary effects. Moreover, p300 or STAT3 overexpression increased MMP19 level, while p300 or STAT3 gene knockdown received opposite results. However, MMP19 expression up-regulation or down-regulation did not alter p300 or STAT3 level, supporting that MMP19 is a downstream effector of p300 and STAT3 in H1299 cells upon IL-17 stimulation.

It has been known that STAT3 and p300 can contribute to various cancer progression [18–22,39–42], and STAT3 activation (e.g., p-STAT3) can co-localize with MMP1, governing MMP1 induction in NSCLC [15]. Meanwhile, activated-STAT3 also can regulate MMP2 gene transcription via binding to MMP2 promoter in esophageal squamous cell carcinoma (ESCC), leading to ESCC metastasis [40]. Besides, p300 can enhance cell migration and invasion by inducing EMT in NSCLC [32] or elevating MMP production by forming EYA3-SIX5-p300 complex accelerating CRC growth and metastasis [41]. Because of a limited understanding of how IL-17, STAT3 and p300 affect MMP19 gene activation, we checked the roles of IL-17, IL-17RA, STAT3 or p300 in MMP19 promoter activity. Our data manifested that IL-17 stimulation, STAT3 or p300 overexpression could elevate MMP19 promoter activity, while IL-17RA, STAT3 or p300 gene knockdown received the contrary effects in H1299 cells exposed to IL-17. Notably, this research revealed that activated-STAT3 could bind to the −544 to −389 nt of MMP19 promoter, and p300 could also bind to the same site of MMP19 promoter in a STAT3-dependent manner, indicating that STAT3 and p300 as a complex can co-localize the specific region of MMP19 promotor promoting its gene transcription.

Recently, documents have reported that p300 can acetylate histones and transcriptional factors regulating cancer progression [31,38,41,42]. Growing evidence has exhibited that acetylated-STAT3 can boost its transcriptional activity via facilitating their access to the DNA template [43], and finally promote cancer metastasis [44]. Since our former results showed p300 and STAT3 could bind to the same element of MMP19 promoter, whether p300 can interact with STAT3 and acetylate it resulting in the changes of aforementioned indicators requires to be identified. Toward this end, we performed assays and verified that IL-17 stimulation, p300 overexpression, or IL-17RA and p300 gene knockdown or p300 activity inhibition could up-regulate or down-regulate the interaction of p300 and STAT3 as well as the level of Ack-STAT3 and p-STAT3. Furthermore, mass spectrometry detection displayed that STAT3-K97, -K354, -K601 and -K631 were all acetylated in IL-17-treated H1299 cells, and mutated assay indicated that only STAT3-K631 was an effective site of p300-mediated STAT3 acetylation. In addition, ChIP, re-ChIP and ChID tests proved that p300-acetylated STAT3-K631 not only could strengthen STAT3 binding to MMP19 promoter, but also increase p-STAT3 and MMP19 expression as well as cell migration and invasion in H1299 cells, while STAT3-K631 mutation (STAT3-K631R) did not show these changes.

Protein can undergo site-specific phosphorylation, acetylation or methylation [23–27,45–47]. Previous studies have confirmed that the canonical IL-6/JAK/STAT3 pathway relies on STAT3-Y705 phosphorylation catalyzed by JAKs, which can induce p-STAT3 translocating into cell nucleus and activate its target gene [23,43,48]. Consistently, STAT3 acetylation belongs to another activating pattern, and Ack-STAT3 in cell nucleus also enhances target gene transcription [27,43,45]. Moreover, the interaction between p-STAT3 and Ack-STAT3 can impact cancer metastasis [44,49]. In this study, although we found that p300, p-STAT3 and Ack-STAT3 were increased in NSCLC tissues and in the cells stimulated with IL-17, and p300 overexpression or knockdown could elevate or reduce the levels of Ack-STAT3, p-STAT3 and MMP19 as well as the cell mobility, whether STAT3 phosphorylation can affect its acetylation and other parameters upon IL-17 has not been clear. Therefore, we treated H1299 cells with Stattic, a p-STAT3-Y705 inhibitor [23,50] or transfected with the mutants of p-STAT3(Y705F) or p-STAT3(Y705D), and displayed that p-STAT3 activity inhibition or p-STAT3(Y705F) transfection could suppress p-STAT3, Ack-STAT3 and MMP19 expression as well as the cell migration and invasion, but p-STAT3(Y705D) transfection possessed the opposite effects, despite p-STAT3(Y705D) did not change Ack-STAT3 level. These data implicate that p300-mediated STAT3-K631 acetylation can increase its Y705-phosphorylation, and STAT3 Y705-phosphorylation also elevates STAT3-K631 acetylation, improving MMP19 production and NSCLC cell migration and invasion via their cooperative interaction. Besides, our animal metastatic model demonstrated that the lung metastatic nodules and MMP19, p-STAT3 or Ack-STAT3 expression were significantly inhibited in the nude mice inoculated with H1299 cells transfected with LV-shp300 or LV-shSTAT3 or LV-shMMP19 plus IL-17 stimulation. Our findings suggest that expression of IL-17-induced p300, Ack-STAT3, p-STAT3 and MMP19 can promote NSCLC cell metastasis.

In summary, our studies found that p300, p-STAT3, Ack-STAT3 and MMP19 were all up-regulated both in NSCLC tissues and in NSCLC cells exposed to IL-17. Functionally, the overexpression or knockdown of p300, STAT3 or MMP19 gene could increase or decrease NSCLC cell migration and invasion. Mechanistically, IL-17-induced p300 and STAT3 as two upstream molecules can bind to −544 to −389 nt of MMP19 gene promoter. In the process, p300 acetylated STAT3-K631 enhanced STAT3 transcriptional activity, Ack-STAT3, p-STAT3 and MMP19 expression as well as cell migration and invasion. Importantly, STAT3-K631 acetylation by p300 and STAT3-Y705 phosphorylation could interact, synergistically promoting MMP19 gene transcription (Fig. 4G). Additionally, the lung metastatic nodules, and MMP19, Ack-STAT3, p-STAT3 expression in metastatic tissues were greatly lessened after the nude mice were inoculated with H1299 cells by silencing p300, STAT3 or MMP19 gene plus IL-17 stimulation. Collectively, these data reveal that NSCLC cell metastasis is related to IL-17/IL-17RA/p300/STAT3/MMP19 axis, which offers a novel insight for NSCLC metastatic mechanism and potential targets for NSCLC treatment.

## Supplementary Materials

FIGURE S1 The mRNA or protein level of selected genes in the cancer tissue of NSCLC patients. (A-C) Total RNA was extracted from the cancer or adjacent tissues of 10 patients with NSCLC, and the mRNA levels of IL-17, IL-17RA (A), or MMP1, MMP2, MMP7, MMP9, MMP11, MMP13, MMP15, MMP19 (B), and Tip60, KAT7, KAT8, PCAF, p300 (C) were detected by RT-PCR. (D and E) The protein samples of 10 pairs of NSCLC and adjacent tissues were respectively mixed, and the expressions of MMP9, MMP19, p300, IL-17RA (D) were examined by IB, so were the expressions of STAT family members (E) and their phosphorylation levels. (F) The acetylation levels of STAT1 and STAT3 were measured by IP using anti-Ack Ab, followed by IB. *pμ0.05, **pμ0.01 vs. adjacent tissues. Data from three independent experiments are expressed as means ± SD, and analyzed by t-test (A-F).

FIGURE S2 Protein expression of IL-17RA, p300, p-STAT3 and MMP19 from the microarrays of 52 NSCLC tissues and paired cancer-adjacent tissues by immunohistochemical (IHC) staining and the correlations between these proteins. (A) Representative images of IL-17RA, p300, p-STAT3 and MMP19 expression stained by IHC (scale bar: 100μm). (B) Statistical analysis of IHC staining scores by intensity and area of the above-mentioned proteins (n=52, **pμ0.01 vs. adjacent tissues). (C) Correlations between IL-17RA, p300, p-STAT3 and MMP19 staining scores were assessed using Pearson's correlation analysis, and the correlation coefficient (R2) was computed. Data are analyzed by t-test (B).

FIGURE S3 Detection of p300, p-STAT3, STAT3, MMP19 expression in BEAS-2B, H1299, PC9, H1975 cell lines (A) and selection of optimal concentration and time for IL-17 stimulation to induce NSCLC cell migration and invasion. (B and C) Change of PC9 cell migration and invasion stimulated by various doses of IL-17 was assessed by wound-healing (B) and Transwell (C) assay (*pμ0.05, **pμ0.01 vs. 0ng/mL). (D) Efficiency of IL-17RA gene knockdown in H1299 cells treated with siIL-17RA. (E and F) Cell migration of H1299 (E) and PC9 (F) cells stimulated by 50ng/mL IL-17 for different time was evaluated by wound-healing experiment (**pμ0.01 vs. 0h). Representative images or data from triplicate experiments are presented as means ± SD and analyzed by one-way ANOVA (B, C, E, F).

FIGURE S4 The effects of IL-17 stimulation on the mRNA levels of selected five transcription factors, five acetyltransferases and five MMP members, and IL-17RA gene knockdown on the mRNA levels of p300 and MMP19 in IL-17-induced H1299 cells. (A-C) H1299 cells were stimulated by 50ng/mL IL-17 for different time, and the mRNA levels of selected five transcription factors (A), five acetyltransferases (B) and five MMP members (C) were detected by RT-PCR (*pμ0.05, **pμ0.01 vs. 0h). (D) Levels of p300 and MMP19 mRNA of H1299 cells exposed to 50ng/mL IL-17 for various time were further examined by real-time PCR (*pμ0.05, **pμ0.01 vs. 0h). (E) H1299 cells were transfected with siIL-17RA for 48h followed by 50ng/mL IL-17 stimulation for 3h, and then the mRNA levels of p300 and MMP19 were measured by real-time PCR (**pμ0.01 vs. DMEM; ▲*p*μ0.05, ▲▲*p*μ0.01 vs. siCTR+IL-17). Data from triplicate experiments are displayed as means ± SD and analyzed by one-way ANOVA (A-E).

FIGURE S5 Verification of constructed p300, STAT3 and MMP19 gene overexpression and knockdown plasmids for the corresponding protein expression. (A-C) H1299 cells were transfected with overexpressed pcDNA3.1/p300 (A), pCMV/STAT3 (B) and pIRES2/MMP19 (C) plasmids for 48h, the protein levels of p300, STAT3 and MMP19 were detected by IB. (D) Transfection efficiency of shCTR was assessed by the GFP positive rate (scale bar: 200μm). (E-G) The expression levels of p300, STAT3 and MMP19 in H1299 cells transfected with shp300 (E), shSTAT3 (F) and shMMP19 (G) for 48h plus 50ng/mL IL-17 stimulation for 3h were identified by IB.

FIGURE S6 Construction of MMP19 full-length (pGL3-MMP19-FL) promoter plasmids, and the effects of IL-17 stimulation, p300 or STAT3 gene overexpression or knockdown on MMP19 promoter activity. (A) PCR of E. coli liquid carrying the promoter plasmids. (B) Part of the sequencing result of the promoter plasmids. (C) H1299 cells were transfected with pGL3-MMP19-FL and the internal control (pRL-SV40) plasmids for 48h or the cells were transfected with siIL-17RA and pGL3-MMP19-FL plasmids for 48h followed by 50ng/mL IL-17 stimulation for 3h, the promoter activity was assessed by luciferase reporter assay (**pμ0.01 vs. DMEM; ▲▲*p*μ0.01 vs. siCTR+IL-17). (D and E) pGL3-MMP19-FL and pRL-SV40 were co-transfected with pcDNA3.1/p300 or pCMV/STAT3 plasmids (D) into H1299 cells for 48h, or with shp300 or shSTAT3 plasmids (E) for 48h plus 50ng/mL IL-17 for 3h, the MMP19 promoter activity was examined by luciferase reporter assay (*pμ0.05 vs. pcDNA3.1; ▲▲*p*μ0.01 vs. shCTR; ##*p*μ0.01 vs. shCTR+IL-17). Data from triplicate experiments are shown as means ± SD and analyzed by one-way ANOVA (C-E).

## Data Availability

The original data supporting the conclusions of our article will be available from the corresponding author on reasonable request.

## Supplementary Materials

The supplementary material is available online at https://doi.org/10.32604/or.2023.031053.
