# Supplementary figures and images for: IL-17 induces NSCLC cell migration and invasion by elevating MMP19 gene transcription and expression through the interaction of p300-dependent STAT3-K631 acetylation and its Y705-phosphorylation
Source: Oncol Res. 2024 Mar 20;32(4):625–41. doi: 10.32604/or.2023.031053 (PMC10972722; doi:10.32604/or.2023.031053)

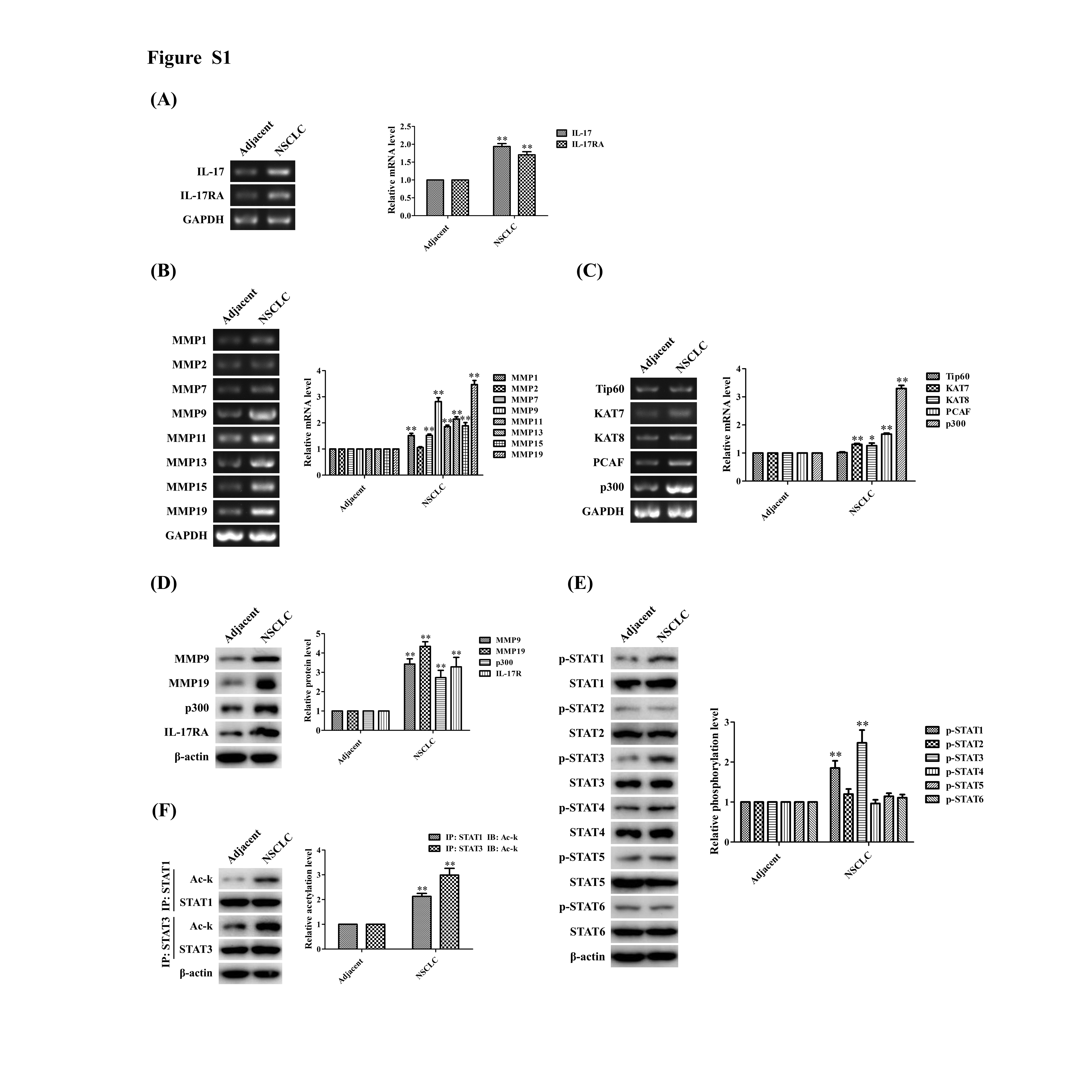

Supplement: FIGURE S1 [file OncolRes-32-31053-s001.tif]

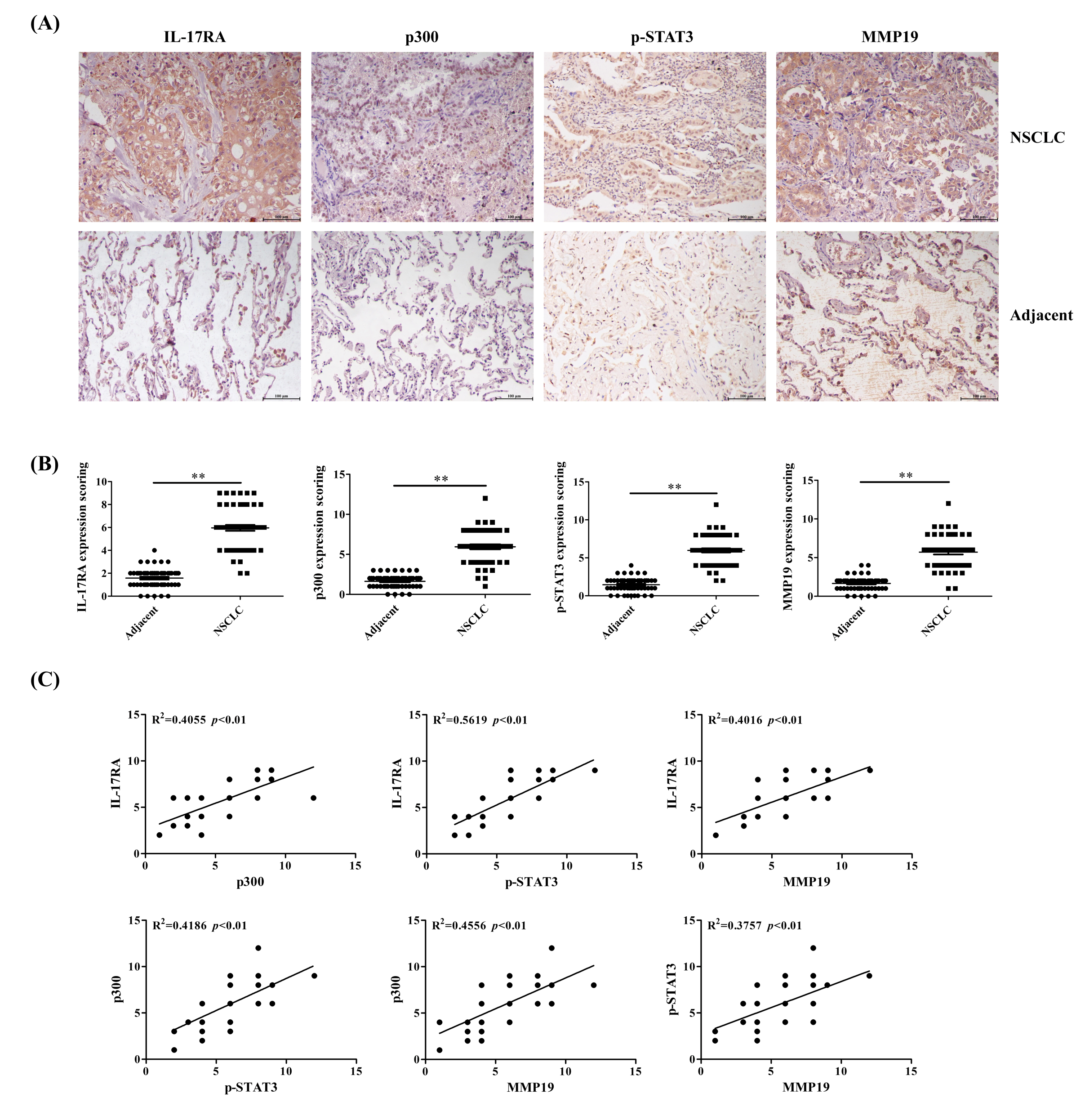

Supplement: FIGURE S2 [file OncolRes-32-31053-s002.tif]

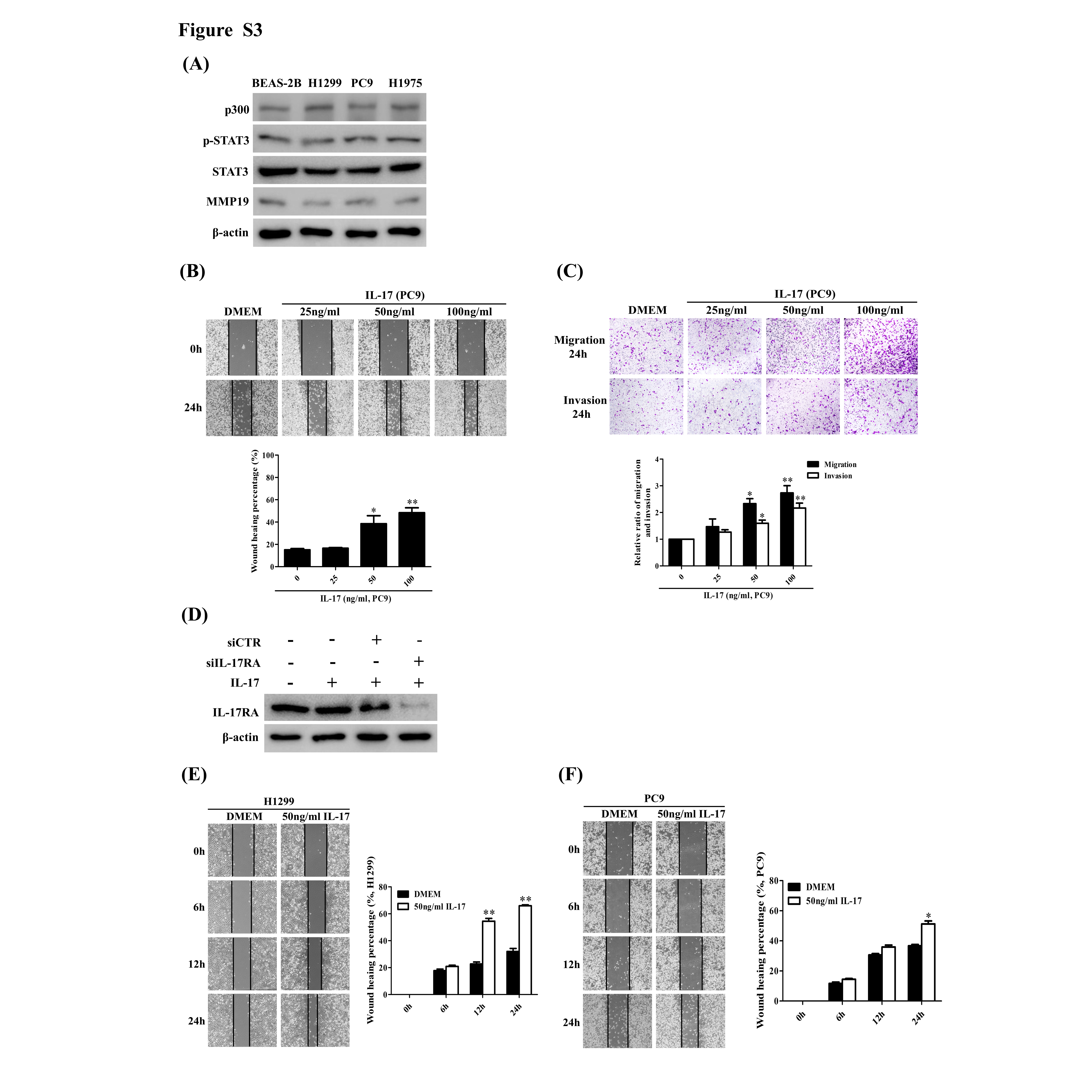

Supplement: FIGURE S3 [file OncolRes-32-31053-s003.tif]

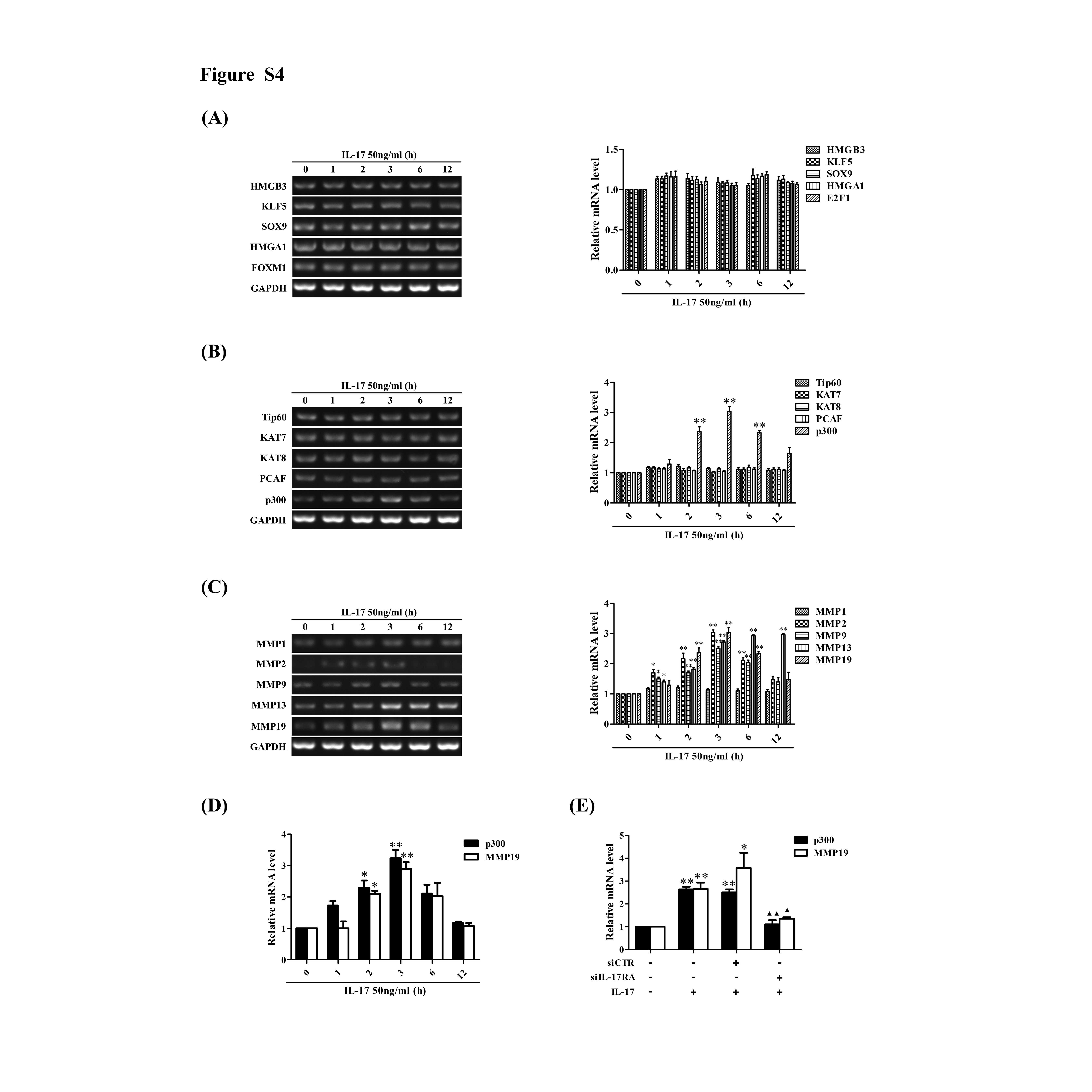

Supplement: FIGURE S4 [file OncolRes-32-31053-s004.tif]

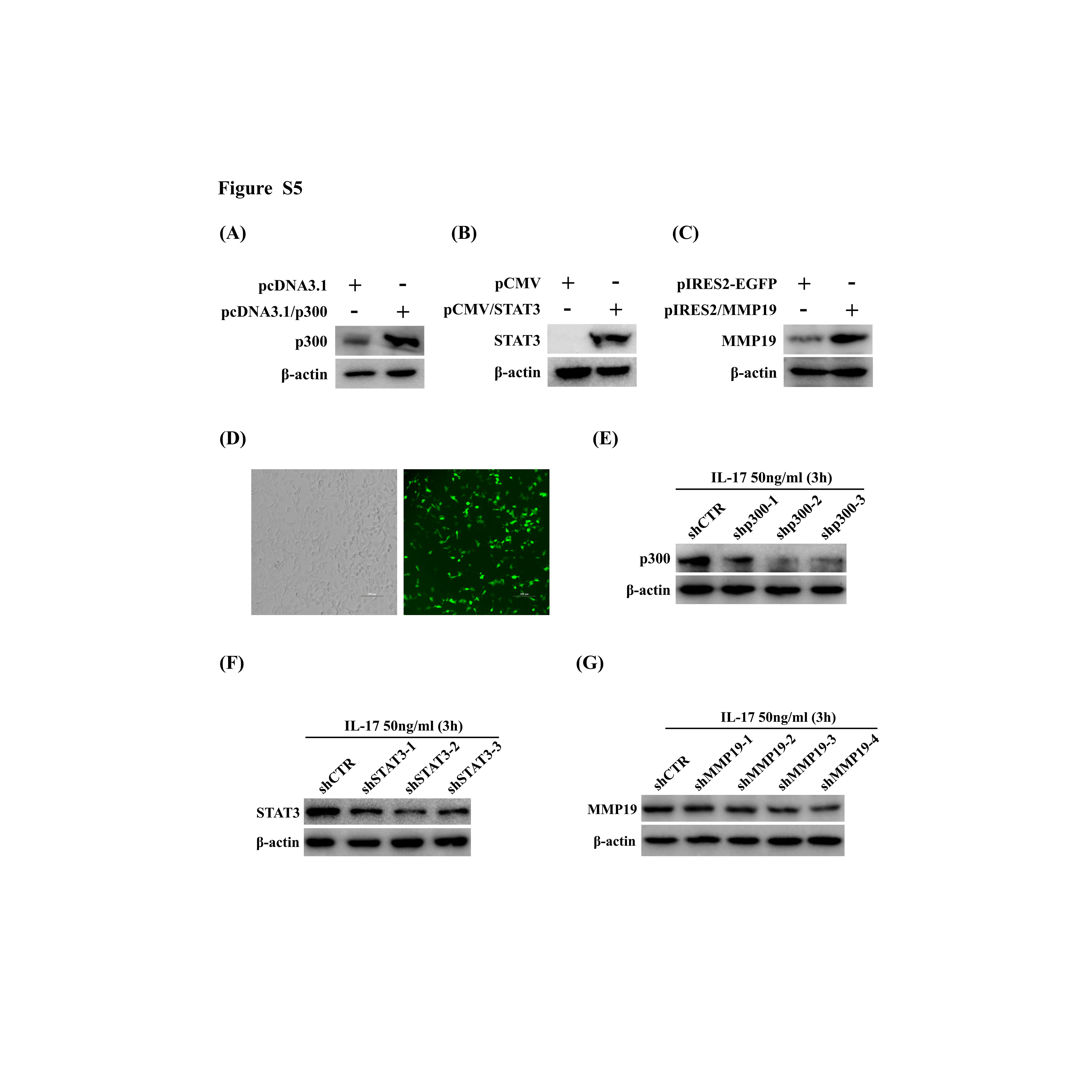

Supplement: FIGURE S5 [file OncolRes-32-31053-s005.tif]

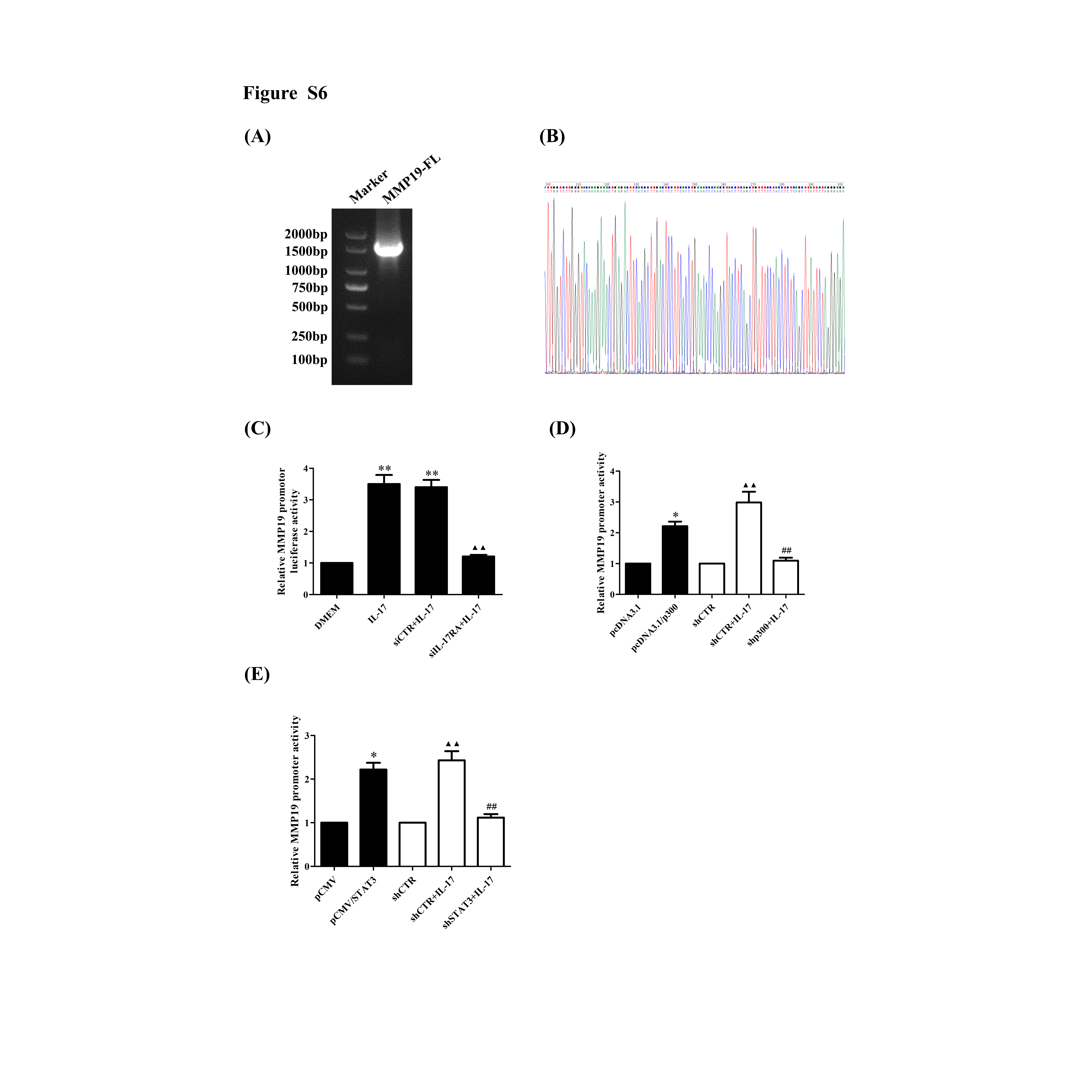

Supplement: FIGURE S6 [file OncolRes-32-31053-s006.tif]
