# Supplementary material for: IL-17 induces NSCLC cell migration and invasion by elevating MMP19 gene transcription and expression through the interaction of p300-dependent STAT3-K631 acetylation and its Y705-phosphorylation
Source: Oncol Res. 2024 Mar 20;32(4):625–41. doi: 10.32604/or.2023.031053 (PMC10972722; doi:10.32604/or.2023.031053)
Supplement: Supplementary file 7 [file OncolRes-32-31053-s001.docx]

**Supplementary Table 1**  The primers of RT-PCR

| Name | Primer | Sequence, 5’→3’ |
| --- | --- | --- |
| IL-17 | Forward | AACTTCCCCCGGACTGTGAT |
|  | Reverse | TGGTAGTCCACGTTCCCATC |
| IL-17RA | Forward | GAGCACATGCACCACATACCT |
|  | Reverse | AGGCCCCGGAATTGGTTCT |
| HMGB3 | Forward | ACATAGGCAAGGTTCAGCAG |
|  | Reverse | GTGTTTCCAAGTAGGAGCAA |
| KLF5 | Forward | GACAGTGCCTCAGTCGTAGAC |
|  | Reverse | TAGATCCGGTGTATTCAGTAGC |
| SOX9 | Forward | CCTCAGGCTTTGCGATTT |
|  | Reverse | GCTCGGGCACTTATTGG |
| HMGA1 | Forward | GCCAACACCTAAGAGACCTC |
|  | Reverse | GCGGCTAAGTGGGATGTT |
| FOXM1 | Forward | TGGGAGGAAATGCCACACTTAG |
|  | Reverse | TAGGACTTCTTGGGTCTTGGGGTG |
| Tip60 | Forward | GAAGATGGCGGAGGTGGTGA |
|  | Reverse | GCTCCTTGGGCAGGTTGAA |
| KAT7 | Forward | GAATGCAAGGTGAGAGCACA |
|  | Reverse | CCGTGTGTTCCCATAGGTCT |
| KAT8 | Forward | GTCACTTCCCTTCCCGCGAT |
|  | Reverse | TCATCATGCTTGCGCTTTTGG |
| PCAF | Forward | TTGCTTCGCTGGGTCTT |
|  | Reverse | CTGGTTGAGGGAATTGCC |
| p300 | Forward | TCCAAGGGGAGAGCGTT |
|  | Reverse | CAGGGCTTTGGTTCGGTAT |
| MMP1 | Forward | GCTGAAAGTGACTGGGAAAC |
|  | Reverse | GGCAAATCTGGCGTGTAA |
| MMP2 | Forward | TGACGGTAAGGACGGACTC |
|  | Reverse | TGGAAGCGGAATGGAAAC |
| MMP7 | Forward | TGGAATGTTAAACTCCCGCGT |
|  | Reverse | GGGATCTCTTTGCCCCACAT |
| MMP9 | Forward | TCCCTGGAGACCTGAGAAC |
|  | Reverse | GATACCCGTCTCCGTGCT |
| MMP11 | Forward | ATGAATTTGGCCACGTGCTG |
|  | Reverse | GCGGTGCAATCTCATTGGTG |
| MMP13 | Forward | GCAGTCTTTCTTCGGCTTAG |
|  | Reverse | AGGGTCCTTGGAGTGGTC |
| MMP15 | Forward | AGAACTGGCTGCGGCTTTAT |
|  | Reverse | TTGGCTTTCACTCGTACCCC |
| MMP19 | Forward | CCTGGACAAATGGGAGTGAC |
|  | Reverse | TTCGAGGCGAGTAGACAGC |
| GAPDH | Forward | CAAGGTCATCCATGACAACTTTG |
|  | Reverse | GTCCACCACCCTGTTGCTGTAG |

**Supplementary Table 2** The primers of real-time PCR and ChIP-PCR

| Name | Primer | Sequence, 5’→3’ |
| --- | --- | --- |
| p300 | Forward | GGGACTCAGCACCGATAACTCA |
|  | Reverse | ACTGCACAGTTCTTATGTGTTCCAA |
| MMP19 | Forward | CCTGGACAAATGGGAGTGAC |
|  | Reverse | TTCGAGGCGAGTAGACAGC |
| β-actin | Forward | CAGCCATGTACGTTGCTATCCAGG |
|  | Reverse | AGGTCCAGACGCAGGATGGCATG |
| MMP19（-564~ -408nt） | Forward | TTACTTATTTGTGCCCTTGTT |
|  | Reverse | GAAGTCTCAGTTTCTTTGTACC |
| MMP19（-544~ -389nt） | Forward | TAAGTCCCACCTACACCCAC |
|  | Reverse | AGGTGAAGGAGTCAAGCGT |

**Supplementary Table 3** The primers of promoter plasmids

| Name | Sequence (5’→3’) |  |
| --- | --- | --- |
| pGL3-MMP19-FL | |  |
| Forward | 5'-GGGGTACCGGTGAATGAAGCAGTCGTG-3' |  |
| Reverse | 5'-GAAGATCTGGGGCAGAGAGAGCAAG-3' |  |
| pGL3-MMP19-1 | |  |
| Forward  Reverse | 5'-GGGGTACCGAATCACCTCTGGCCTG-3'  5'-GAAGATCTGGGGCAGAGAGAGCAAG-3' |  |
| pGL3-MMP19-2 | |  |
| Forward  Reverse | 5'-GGGGTACCTGCTGGCCCAGGGTC-3'  5'-GAAGATCTGGGGCAGAGAGAGCAAG-3' |  |
| pGL3-MMP19-3 | |  |
| Forward  Reverse | 5'-GGGGTACCTAATGGTGAATTGCAACATA-3'  5'-GAAGATCTGGGGCAGAGAGAGCAAG-3' |  |
| pGL3-MMP19-4 | |  |
| Forward  Reverse | 5'-GGGGTACCGTCAGAGGCACGGACA-3'  5'-GAAGATCTGGGGCAGAGAGAGCAAG-3' |  |

**Supplementary Table 4**  The correlation between IL-17RA expression and clinic-pathological features in 52 cases of NSCLC patients

| Characteristics | Total | IL-17RA expression | | *p* value^#^ |
| --- | --- | --- | --- | --- |
|  |  | Weak and negative | Strong |  |
|  | 52 | 15 | 37 |  |
| Sex |  |  |  |  |
| Male | 34 | 9 (60%) | 25 (67.57%) | 0.6033 |
| Female | 18 | 6 (40%) | 12 (32.43%) | |
|  |  |  |  |  |
| Age (years) |  |  |  |  |
| <60 | 28 | 7 (46.67%) | 21 (56.76%) | 0.5085 |
| ≥60 | 24 | 8 (53.33%) | 16 (43.24%) | |
|  |  |  |  |  |
| Tumor size |  |  |  |  |
| <5 cm | 35 | 10 (66.67%) | 25 (67.57%) | 0.9500 |
| ≥5 cm | 17 | 5 (33.33%) | 12 (32.43%) | |
|  |  |  |  |  |
| Lymph node metastasis |  |  |  |  |
| Negative | 14 | 8 (53.33%) | 6 (16.22%) | 0.0063* |
| Positive | 38 | 7 (46.67%) | 31 (83.78%) | |
|  |  |  |  |  |
| TNM stage |  |  |  |  |
| I+II | 32 | 15 (100%) | 17 (53.13%) | 0.0013* |
| III | 15 | 0 (0%) | 15 (46.88%) | |
|  |  |  |  |  |
| Pathologic type |  |  |  |  |
| Adenocarcinoma | 26 | 9 (60%) | 17 (45.95%) | 0.4845 |
| Squamous carcinoma | 14 | 4 (26.67%) | 10 (27.03%) | |
| Adenosquamous carcinoma | 7 | 2 (13.33%) | 5 (13.51%) |  |
| Large cell carcinoma | 5 | 0 (0%) | 5 (13.51%) |  |

^#^chi-square test

**p*<0.05

**Supplementary Table 5** The correlation between p300 expression and clinic-pathological features in 52 cases of NSCLC patients

| Characteristics | Total | p300 expression | | *p* value^#^ |
| --- | --- | --- | --- | --- |
|  |  | Weak and negative | Strong |  |
|  | 52 | 16 | 36 |  |
| Sex |  |  |  |  |
| Male | 34 | 10 (62.5%) | 24 (66.67%) | 0.7707 |
| Female | 18 | 6 (37.5%) | 12 (33.33%) | |
|  |  |  |  |  |
| Age (years) |  |  |  |  |
| <60 | 28 | 8 (50%) | 20 (55.56%) | 0.7107 |
| ≥60 | 24 | 8 (50%) | 16 (44.44%) | |
|  |  |  |  |  |
| Tumor size |  |  |  |  |
| <5 cm | 35 | 11 (68.75%) | 24 (66.67%) | 0.8825 |
| ≥5 cm | 17 | 5 (31.25%) | 12 (33.33%) | |
|  |  |  |  |  |
| Lymph node metastasis |  |  |  |  |
| Negative | 14 | 10 (62.5%) | 4 (11.11%) | 0.0001* |
| Positive | 38 | 6 (37.5%) | 32 (88.89%) | |
|  |  |  |  |  |
| TNM stage |  |  |  |  |
| I+II | 32 | 14 (100%) | 18 (54.55%) | 0.0022* |
| III | 15 | 0 (0%) | 15 (45.45%) | |
|  |  |  |  |  |
| Pathologic type |  |  |  |  |
| Adenocarcinoma | 26 | 12 (75%) | 14 (38.89%) | 0.0538 |
| Squamous carcinoma | 14 | 1 (6.25%) | 13 (36.11%) | |
| Adenosquamous carcinoma | 7 | 1 (6.25%) | 6 (16.67%) |  |
| Large cell carcinoma | 5 | 2 (12.5%) | 3 (8.33%) |  |

^#^chi-square test

**p*<0.05

**Supplementary Table 6**  The correlation between p-STAT3 expression and clinic-pathological features in 52 cases of NSCLC patients

| Characteristics | Total | p-STAT3 expression | | *p* value^#^ |
| --- | --- | --- | --- | --- |
|  |  | Weak and negative | Strong |  |
|  | 52 | 16 | 36 |  |
| Sex |  |  |  |  |
| Male | 34 | 8 (50%) | 26 (72.22%) | 0.1200 |
| Female | 18 | 8 (50%) | 10 (27.78%) | |
|  |  |  |  |  |
| Age (years) |  |  |  |  |
| <60 | 28 | 9 (56.25%) | 19 (52.78%) | 0.8167 |
| ≥60 | 24 | 7 (43.75%) | 17 (47.22%) | |
|  |  |  |  |  |
| Tumor size |  |  |  |  |
| <5 cm | 35 | 11 (68.75%) | 24 (66.67%) | 0.8825 |
| ≥5 cm | 17 | 5 (31.25%) | 12 (33.33%) | |
|  |  |  |  |  |
| Lymph node metastasis |  |  |  |  |
| Negative | 14 | 8 (50%) | 6 (16.67%) | 0.0124* |
| Positive | 38 | 8 (50%) | 30 (83.33%) | |
|  |  |  |  |  |
| TNM stage |  |  |  |  |
| I+II | 32 | 15 (100%) | 17 (53.13%) | 0.0013* |
| III | 15 | 0 (0%) | 15 (46.88%) | |
|  |  |  |  |  |
| Pathologic type |  |  |  |  |
| Adenocarcinoma | 26 | 11 (68.75%) | 15 (41.67%) | 0.2959 |
| Squamous carcinoma | 14 | 2 (12.5%) | 12 (33.33%) | |
| Adenosquamous carcinoma | 7 | 2 (12.5%) | 5 (13.89%) |  |
| Large cell carcinoma | 5 | 1 (6.25%) | 4 (11.11%) |  |

^#^chi-square test

**p*<0.05

**Supplementary Table 7** The correlation between MMP19 expression and clinic-pathological features in 52 cases of NSCLC patients

| Characteristics | Total | MMP19 expression | | *p* value^#^ |
| --- | --- | --- | --- | --- |
|  |  | Weak and negative | Strong |  |
|  | 52 | 19 | 33 |  |
| Sex |  |  |  |  |
| Male | 34 | 13 (68.42%) | 21 (63.64%) | 0.7269 |
| Female | 18 | 6 (31.58%) | 12 (36.36%) | |
|  |  |  |  |  |
| Age (years) |  |  |  |  |
| <60 | 28 | 9 (47.37%) | 19 (57.58%) | 0.4771 |
| ≥60 | 24 | 10 (52.63%) | 14 (42.42%) | |
|  |  |  |  |  |
| Tumor size |  |  |  |  |
| <5 cm | 35 | 14 (73.68%) | 21 (63.64%) | 0.4570 |
| ≥5 cm | 17 | 5 (26.32%) | 12 (36.36%) | |
|  |  |  |  |  |
| Lymph node metastasis |  |  |  |  |
| Negative | 14 | 10 (52.63%) | 4 (12.12%) | 0.0015* |
| Positive | 38 | 9 (47.37%) | 29 (87.88%) | |
|  |  |  |  |  |
| TNM stage |  |  |  |  |
| I+II | 32 | 16 (94.12%) | 16 (53.33%) | 0.0040* |
| III | 15 | 1 (5.88%) | 14 (46.67%) | |
|  |  |  |  |  |
| Pathologic type |  |  |  |  |
| Adenocarcinoma | 26 | 13 (68.42%) | 13 (39.39%) | 0.1551 |
| Squamous carcinoma | 14 | 2 (10.53%) | 12 (36.36%) | |
| Adenosquamous carcinoma | 7 | 2 (10.53%) | 5 (15.15%) |  |
| Large cell carcinoma | 5 | 2 (10.53%) | 3 (9.09%) |  |

^#^chi-square test

**p*<0.05
